# Identification of Milk and Cheese Intake Biomarkers in Healthy Adults Reveals High Interindividual Variability of Lewis System–Related Oligosaccharides

**DOI:** 10.1093/jn/nxaa029

**Published:** 2020-03-04

**Authors:** Grégory Pimentel, David Burnand, Linda H Münger, François P Pralong, Nathalie Vionnet, Reto Portmann, Guy Vergères

**Affiliations:** 1 Federal Department of Economic Affairs, Education, and Research, Agroscope, Bern, Switzerland; 2 Service of Endocrinology, Diabetes, and Metabolism, Lausanne University Hospital, Lausanne, Switzerland

**Keywords:** biomarker, dairy, cheese, milk, soy, postprandial, metabolomics, serum metabolome, Lewis antigen

## Abstract

**Background:**

The use of biomarkers of food intake (BFIs) in blood and urine has shown great promise for assessing dietary intake and complementing traditional dietary assessment tools whose use is prone to misreporting.

**Objective:**

Untargeted LC-MS metabolomics was applied to identify candidate BFIs for assessing the intake of milk and cheese and to explore the metabolic response to the ingestion of these foods.

**Methods:**

A randomized controlled crossover study was conducted in healthy adults [5 women, 6 men; age: 23.6 ± 5.0 y; BMI (kg/m^2^): 22.1 ± 1.7].  After a single isocaloric intake of milk (600 mL), cheese (100 g), or soy-based drink (600 mL), serum and urine samples were collected postprandially up to 6 h and after fasting after 24 h. Untargeted metabolomics was conducted using LC-MS. Discriminant metabolites were selected in serum by multivariate statistical analysis, and their mass distribution and postprandial kinetics were compared.

**Results:**

Serum metabolites discriminant for cheese intake had a significantly lower mass distribution than metabolites characterizing milk intake (*P* = 4.1 × 10^−4^). Candidate BFIs for milk or cheese included saccharides, a hydroxy acid, amino acids, amino acid derivatives, and dipeptides. Two serum oligosaccharides, blood group H disaccharide (BGH) and Lewis A trisaccharide (LeA), specifically reflected milk intake but with high interindividual variability. The 2 oligosaccharides showed related but opposing trends: subjects showing an increase in either oligosaccharide did not show any increase in the other oligosaccharide. This result was confirmed in urine.

**Conclusions:**

New candidate BFIs for milk or cheese could be identified in healthy adults, most of which were related to protein metabolism. The increase in serum of LeA and BGH after cow-milk intake in adults calls for further investigations considering the beneficial health effects on newborns of such oligosaccharides in maternal milk. The trial is registered at clinicaltrials.gov as NCT02705560.

## Introduction

The validity of nutritional studies relies on an accurate assessment of participants’ food intake and dietary exposure. However, traditional tools of dietary assessment such as 24-h recalls, food-frequency questionnaires or food records are prone to under-/overestimation bias as they rely on subjects to self-report (1–3). Biomarkers of food intake (BFIs) have shown great promise as a tool for addressing this issue. BFIs are compounds measured within the body that accurately reflect the intake of a food or food constituent (4, 5). BFIs can be considered as unbiased and objective surrogate estimators of food intake, but also as a tool to evaluate metabolic status and to highlight associations between diet and metabolic disorders (2, 6). For the discovery of BFIs, the Joint Programming Initiative “A Healthy Diet for a Healthy Life” Food Biomarkers Alliance (FoodBAll) has conducted standardized intervention studies consisting of postprandial sampling after an acute intake of a specific test food, completed with 24-h fasting sampling (6). The consortium opted for an untargeted metabolomics analysis of urine and serum samples as this method allows the simultaneous measurement of a large number of low-molecular-weight metabolites. Using this approach, the consortium has successfully identified candidate BFIs for several foods, including cereals (7), fruits (8, 9), meat (10), peas (11), and milk and cheese (12, 13). These studies highlighted the importance of the postprandial phase for the detection of candidate BFIs, as well as the challenges particular to the validation of BFIs (i.e., the lack of specificity for the tested food and the confirmation of the observed postprandial trends in the fasting state), and therefore the necessity to validate results in cohort studies under free-living conditions (6, 14). Few nutritional interventions have aimed to identify BFIs for dairy products despite their prominent place in Western diets and their widely discussed effects on health. A systematic review published by members of the FoodBAll consortium listed and evaluated putative BFIs for dairy, including metabolites related to lipid (C17:0, C:15:0, C17:1, myristoyl-sphingomyelin or methyl palmitate), galactose (galactonate), or amino acid metabolism for cheese intake (15). However, most of the reviewed studies were not specifically designed for BFI discovery and the authors recommended further investigation to validate the proposed biomarkers. Within the framework of the FoodBAll project, an acute intervention study focused on the discovery of dairy BFIs. GC-MS and NMR analytical platforms characterized milk intake by an increase in lactose-derived compounds (lactose, galactose, galactitol, galactonate, and galactono-1,5-lactone), while most BFIs for cheese were related to protein and amino acid metabolism (3-phenyllactic acid, methionine, proline, leucine, tyrosine, valine, and isoleucine) (12, 13).

In addition to BFI identification, postprandial untargeted metabolomics can comprehensively characterize the specific metabolic response of individuals to the ingestion of a test food. The interindividual variability observed in the postprandial phase can be used to *1*) estimate the capacity of a subject to respond to a dietary challenge and thus evaluate overall metabolic health (16, 17), *2*) identify dysregulation of metabolic pathways that is not necessarily visible in the fasting state (18, 19), and *3*) identify metabolic subgroups of subjects according to their response to the ingestion of a test food and further investigate these groups in the context of other nutrigenomic tools (20, 21).

This report presents results obtained from the FoodBAll nutritional intervention for dairy products for serum and urine samples analyzed by LC-MS–based metabolomics. By covering a specific spectrum of the metabolome, LC-MS analysis aimed to identify new putative BFIs for milk or cheese (compared with a soy drink as a nondairy control), and therefore complement the previous results obtained by GC-MS and NMR (12, 13). By further characterizing the postprandial kinetics and interindividual variability of BFIs, we evaluated their potential to reflect not only food intake but also metabolic phenotypes.

## Methods

### Study design

The present project was conducted within the framework of the FoodBAll project. Given the exploratory nature of the untargeted metabolomics analysis, the desired sample size for the study could not be calculated a priori and is therefore based on similar published crossover nutritional studies that used untargeted metabolomics analyses on postprandial samples. A sample size in the range of 10–15 subjects has been shown to give sufficient statistical power to identify regulated metabolites and, in particular, potential BFIs (6, 10, 22, 23). The study was a controlled, crossover study and conducted in 11 participants (5 women and 6 men; **Supplemental Figure 1**). The subjects were regular consumers of dairy products, aged 19–31 y, and with a BMI (in kg/m^2^) ranging between 18 and 30. Details regarding exclusion and inclusion criteria and characteristics of study population are given by Münger et al. (12). The study was conducted in accordance with the Declaration of Helsinki, received ethical approval from the Commission Cantonale d'Ethique de la Recherche sur l'Etre Humain (Vaud, Switzerland), and was registered at clinicaltrials.gov as NCT02705560. All subjects provided written informed consent beforehand.

**FIGURE 1:**
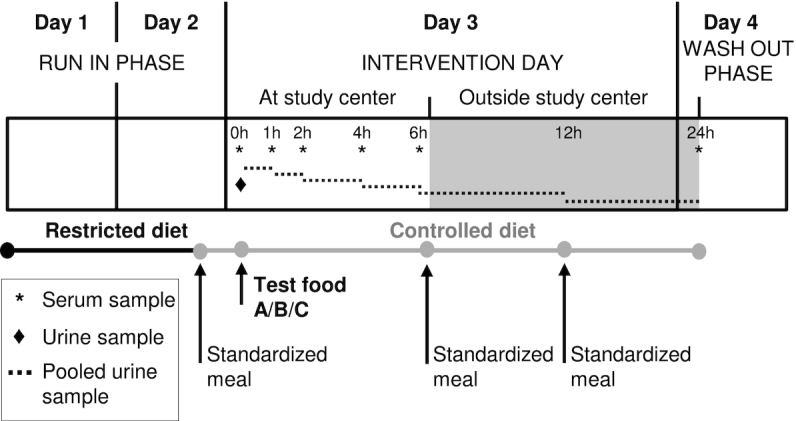
Overview of the controlled, crossover study design. During the intervention day, 11 healthy subjects ingested isocaloric doses of three test products in random order (A = milk, B = cheese, C = soy drink). Serum and pooled urine samples were collected after an overnight fast and postprandially, up to 6 h for serum and up to 12 h for urine. The following day after fasting, serum and pooled urine samples were collected. The participants followed a 2-d restricted diet before the intervention day and consumed a controlled diet composed of standardized meals during the study.

The study followed the general design defined within the FoodBAll project (**Figure 1**). Briefly, the subjects came to the study center after 12 h of fasting for an intervention day. The 3 foods tested were 600 mL organic pasteurized full fat milk (3.9% fat, Coop Naturaplan, Switzerland), 100 g hard cheese (Le Gruyère AOP, Fromagerie Bullet, Bullet, Switzerland) plus 500 mL of water, and 600 mL soy drink, which was a mixture of soy milk and added vegetable fat (Soja Line, Migros, Switzerland). All 3 test products were isocaloric (∼400 kcal); details regarding their composition are given by Münger et al. (12). Blood samples were taken before (*t* = 0 h) and postprandially after the ingestion of 1 of the 3 test products (*t* = 1, 2, 4, and 6 h). Urine samples were taken postprandially during the time intervals 0−1, 1−2, 2−4, and 4−6 h. After the postprandial phase, urine was collected during the time intervals 6−12 and 12−24 h. The following morning, blood samples were collected under fasting conditions (*t* = 24 h). During the 2-d run-in phase, diet was restricted to non–bovine-, non–dairy-, and non–soy-containing foods. In addition, participants were given standardized meals the evening before and during the intervention day. Details regarding blood and urine sample preparation for LC-MS analysis are given in the **Supplemental Methods**.

### Untargeted LC-MS–based metabolomics analysis

The UltiMate 3000 HPLC system (Thermo Fisher Scientific), coupled with the maXis 4G+ quadrupole time-of-flight mass spectrometer (Bruker Daltonik GmbH) were used for the untargeted metabolomics analysis. The mass spectrometer electrospray interface was operating in positive ion mode and spectra were recorded from *m/z* 75 to *m/z* 1500. Collision-induced dissociation was performed using energies from 20 to 70 eV. Detailed serum and urine sample preparation as well as chromatography and spectrometer settings are given in the Supplemental Methods.

### Data processing and statistical analysis

#### Reduction and filtering of serum and urine datasets

Progenesis QI (v.2.3.6198.24128; NonLinear Dynamics Ltd.) was used for retention time correction, peak-picking, deconvolution, adducts annotation, and normalization (default automatic sensitivity and without minimum peak width). The dataset was corrected to account for signal drift, and reduced via multiple filtering steps to remove metabolites with poor repeatability and potential contaminants (Supplemental Methods). The net incremental AUC (iAUC) was calculated for each metabolite in the postprandial phase using R (v.3.1.2, MESS R package; R Foundation for Statistical Computing). After principal components analysis, a group of samples obtained from one male subject were considered as technical outliers (outside the Hotelling T2 ellipse with 95% CI) and removed from the dataset, resulting in ten subjects remaining out of eleven subjects. The dataset was further reduced by retaining only metabolites that presented a significant postprandial response after ≥1 of the three test foods [nonparametric analysis of longitudinal data (nparLD); R package; 0.05 as the *P*-value significance cutoff] (24). Similarly, a paired Wilcoxon signed-rank test was used to identify and filter for metabolites presenting a significant change in fasting serum concentrations after 24 h (*t* = 0 h vs. *t* = 24 h). The same data-reduction procedure was used on the dataset from urine samples.

#### Selection of discriminant metabolites by multivariate statistical analysis

Partial least-square discriminant analysis (PLS-DA) was performed to differentiate serum samples collected after milk, cheese, and soy drink intake (SIMCA-P software v.14.0; Umetrics). Orthogonal PLS-DA (OPLS-DA) was carried out for 1 by 2 comparisons (milk vs. cheese/soy drink, cheese vs. milk/soy drink, and soy drink vs. cheese/milk). The dataset was scaled using the Pareto method. Validity of the models was evaluated by the goodness-of-fit parameter (R2Y), the predictive ability parameter (Q2; calculated by 10-fold cross-validation, Q2 >0.50 as a cutoff value), ANOVA of the cross-validated residuals to confirm the reliability of the model (CV-ANOVA; *P* < 0.05 as a significance threshold) (25), and permutation tests with 999 random permutations to exclude any random separation of the sample groups (26). Finally, discriminant metabolites were selected based on variable importance in projection (VIP) scores (VIP >1.5 as a cutoff value).

#### Univariate statistical analysis, postprandial kinetics, and mass distribution of discriminant metabolites

For the discriminant metabolites selected by multivariate analysis (VIP score >1.5), the Kruskal-Wallis test was also applied to assess the effect of test foods, at each time point and on the 6-h postprandial iAUC (*P* < 0.05 as the significance threshold). When significant, the Kruskal-Wallis test was completed by a post-hoc Conover-Iman pairwise comparison test. The same approach was used to compare the levels of selected discriminant metabolites in different subgroups of subjects. Metabolites discriminant for milk, cheese, or soy intake were clustered according to their postprandial kinetics after the intake of each of the three foods. Hierarchical clustering analysis was performed using the R *pheatmap* package (version 1.0.12) with maximum distance measure and Ward's linkage. The mass distributions of discriminant metabolites for milk and cheese intake (smoothed densities of *m/z* values using R) were compared using the Kolmogorov-Smirnov test (*P* < 0.05 as the significance threshold).

### Identification of discriminant metabolites

The Human Metabolome Database (27), the MassBank of North America (28), and the National Institute of Standards and Technology database (NIST v14) were used with a 5-ppm mass accuracy threshold for the identification of discriminant metabolites. Identity suggestions from databases were then confirmed by MS fragmentation data (when available) and/or with the injection of pure standards solutions. The list of standards suppliers is given in the Supplemental Methods.

The level of identification of each discriminant metabolite is defined according to the Metabolomics Standards Initiative recommendations (29), as follows—level 1: compounds were identified by comparison to a pure reference based on spectral data (molecular weight with a 5-ppm accuracy threshold, fragmentation pattern when available, isotopic distribution, and retention time with 10% accuracy threshold); level 2: without chemical standards, based on spectral data; level 3: putatively characterized compound classes; and level 4: unknown compound. Discriminant metabolites were considered as putative BFIs if they showed an increase in postprandial or fasting serum collected after the intake of the food of interest. The presence of the putative BFIs selected in serum was investigated in the filtered urine dataset.

## Results

### Serum metabolome postprandial response

Following data filtering, a total of 2988 unique serum metabolites were detected. A total of 1639 metabolites (55%) showed a significant postprandial kinetic after the ingestion of ≥ 1 of the three test foods (nparLD test, nonadjusted *P* < 0.05). Interestingly, one-third of the postprandial metabolome (973 metabolites, 32.6%) showed a postprandial response specifically after the intake of one of the three foods (382 metabolites showed a dynamic response only after the intake of milk, 426 only after cheese intake, and 165 only after soy intake). A total of 430 metabolites (14%) showed a significant difference in fasting serum 24 h after the intake of ≥1 of the test foods (Wilcoxon test, nonadjusted *P* < 0.05). Detailed numbers of metabolites showing a significant postprandial response or significant change in fasting serum are given in **Table 1**.

**TABLE 1:** Serum metabolites presenting a significant postprandial response, or a significant change in 24 h fasting serum after the ingestion of milk, cheese, or soy in healthy adults^1^

	Significant postprandial response^2^		Significant change in 24-h fasting serum^3^	
	Serum metabolites, *n*	% of total	Serum metabolites, *n*	% of total
After ≥1 food	1639	54.9	430	14.4
Including milk	901	30.2	184	6.2
Including cheese	1005	33.6	155	5.2
Including soy	736	24.6	220	7.4
After 1 food only	973	32.6	333	11.1
Milk only	382	12.8	102	3.4
Cheese only	426	14.3	96	3.2
Soy only	165	5.5	135	4.5
After ≥2 foods	666	22.3	98	3.3
Milk and cheese	432	14.5	43	1.4
Milk and soy	424	14.2	69	2.3
Cheese and soy	484	16.2	46	1.5
After 2 foods only	329	11.0	68	2.3
Milk and cheese only	95	3.2	13	0.4
Milk and soy only	87	2.9	39	1.3
Cheese and soy only	147	4.9	16	0.5
After all 3 foods	337	11.3	30	1.0

1 *n*  = 10 subjects. Serum metabolome measured by LC-MS untargeted metabolomics, 2988 unique metabolites detected.

2 Postprandial time effect evaluated by nonparametric longitudinal data analysis (*P* < 0.05 as the significance threshold).

3 Change in fasting serum evaluated by paired Wilcoxon signed-rank test (*P* < 0.05 as the significance threshold).

### Differentiation of serum samples by multivariate statistical analysis

PLS-DA analysis using the selection of 1639 postprandial serum metabolites showed a clear separation of serum samples taken postprandially after milk, cheese, or soy intake (**Supplemental Figure 2**). The model showed good fit and predictability (R2 = 0.88, Q2 = 0.62, CV-ANOVA *P* = 1.3 × 10^−3^).

Postprandial 1-by-2 OPLS-DA comparisons indicated that serum taken after a test food could be differentiated from serum taken after the 2 other foods (milk vs. soy/cheese, cheese vs. milk/soy, or soy vs. milk/cheese). The strongest separation was obtained when comparing serum samples taken after soy drink intake against nonsoy intake (soy vs. milk/cheese) (R2 = 0.95, Q2 = 0.78, CV-ANOVA *P* = 5.3 × 10^−8^). The lowest predictability was observed when comparing milk intake against nonmilk intake (milk vs. cheese/soy) (R2 = 0.88, Q2 = 0.55, CV-ANOVA *P* = 3.4 × 10^−3^) (**Supplemental Figures 3–5**). Conversely, PLS-DA analysis using the selection of 430 metabolites could not differentiate serum samples taken under fasting condition 24 h after the ingestion of milk, cheese, or soy (R2 = 0.36, Q2 = 0.16, CV-ANOVA *P* = 3.8 × 10^−1^; data not shown). OPLS-DA 1-by-2 comparisons showed that only soy intake compared with nonsoy intake led to a difference in 24-h fasting serum, the Q2 value reaching almost the cutoff value of 0.50 (R2 = 0.91, Q2 = 0.45, CV-ANOVA *P* = 8.8 × 10^−3^) (**Supplemental Figure 6**). Serum samples taken 24 h after milk or cheese intake could not be differentiated from samples taken after nonmilk and noncheese intake, respectively (R2 = 0.97, Q2 = 0.37, CV-ANOVA *P* = 2.1 × 10^−1^, and R2 = 0.99, Q2 = 0.31, CV-ANOVA *P* = 5.8 × 10^−1^, respectively; data not shown).

A total of 297 metabolites were considered discriminant (VIP >1.5): 261 in the postprandial phase, including 65 metabolites for milk intake (25%); 124 metabolites for cheese intake (47%); and 72 for soy intake (28%). Forty-five metabolites were considered discriminant for soy intake in 24-h fasting serum.

### Metabolites discriminant for milk, cheese, and soy drink intake showed different postprandial kinetics and mass distributions

**FIGURE 2:**
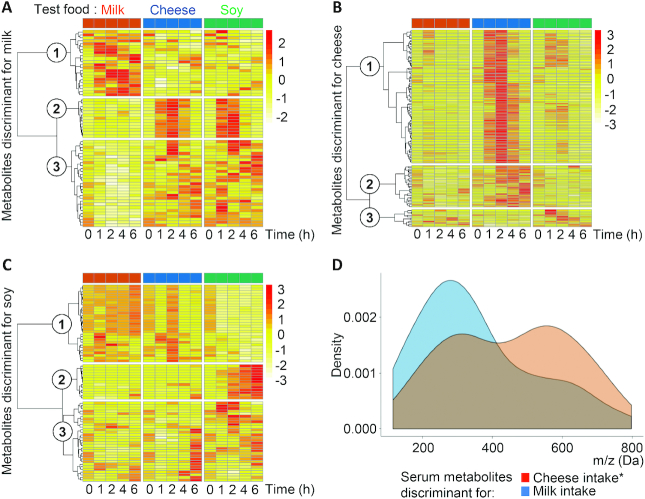
Postprandial kinetics and mass distributions of metabolites discriminant for milk, cheese, or soy drink intake in serum of healthy adults (*n* = 10). Metabolites discriminant for milk (A), cheese (B), and soy drink (C) intake were selected by 1-by-2 OPLS-DA analysis (VIP score >1.5 as the significance threshold). Hierarchical clustering analysis according to postprandial kinetics after the intake of each of the 3 foods was performed using Maximum's distance measure and Ward linkage. The circled numbers 1 to 3 in the figures refer to the cluster numbering. Mass distribution density plots of discriminant metabolites (D) were compared using the Kolmogorov-Smirnov test. *Significant difference between mass distributions (*P* < 0.05). OPLS-DA, orthogonal projections to latent structures discriminant analysis; VIP score, variable importance in projection for the predictive component.

The postprandial kinetics of metabolites discriminant for milk, cheese, or soy drink intake were classified by hierarchical clustering analyses (**Figure 2**). Interestingly, heatmaps showed that metabolites discriminant for milk, cheese, or soy drink intake present different postprandial kinetics. While metabolites discriminant for cheese intake (Figure 2B) are mostly metabolites that increased in serum during the early (cluster 1) or late (cluster 2) postprandial phase, metabolites discriminant for milk intake (Figure 2A) are essentially metabolites that, compared with cheese and soy drink intake, remain unchanged (cluster 2) or decreased (cluster 3) postprandially. Only one-third of the metabolites discriminant for milk intake showed increased iAUCs compared with cheese and soy intake (cluster 1). Most of metabolites discriminant for soy drink intake (Figure 2C) either decreased (cluster 1) or increased only in the late postprandial phase (cluster 2).

In addition, to further characterize the selected BFIs, and in particular the influence of the fermentation step, metabolites discriminant for milk and cheese intake were compared according to their mass distribution. This analysis revealed bimodal distributions of the masses centered at masses (*m/z*) of ∼300 and 600 Da. However, compared with the response after milk intake, the relative distribution of these masses was shifted toward the lower masses after cheese intake. Overall, a statistical analysis of these distributions showed that metabolites discriminant for cheese intake had a significantly lower mass distribution than metabolites discriminant for milk intake (*P* = 4.1 × 10^−4^; Figure 2D).

### Identification of discriminant metabolites and selection of putative BFIs

Of the 297 discriminant metabolites that were selected in postprandial and/or fasting serum, 9 have been identified at level 1, 23 at level 2, and 30 at level 3, and 199 at level 4 remained unknown. **Supplemental Table 1** details all of the discriminant metabolites.

Among the discriminant metabolites with level 1 identification, 8 were considered as putative BFIs (**Table 2**), with 5 also being detected in urine. Two BFIs were specific to milk intake: blood group H disaccharide (2-O-a-L-fucopyranosyl-galactose; BGH) and Lewis A trisaccharide (3-O-b-D-galactopyranosyl-4-O-a-L-fucopyranosyl-N-acetyl-D-glucosamine; LeA). The analytical method used could not differentiate galactonic acid from gluconic acid (stereoisomers) as the standards of both compounds had identical retention time, but this metabolite was specific to milk intake. Six discriminant metabolites were considered as putative BFIs for cheese: aminoadipic acid, citrulline, valyl-threonine (VT), phenylalanyl-proline (FP), indole-3-lactic acid (ILA), and proline. The specificity of aminoadipic acid is, however, limited as it showed a significant postprandial increase after the intake of each of the 3 foods (nparLD, *P* < 0.05). The most important change was observed after cheese intake, especially after 4 h, resulting in a higher iAUC (VIP cheese vs. non-cheese = 1.84).

**TABLE 2:** Identified BFIs for milk or cheese selected by 1-by-2 OPLS-DA analysis in serum of healthy adults^1^

Retention time, min	Adduct mass, Da/charge	Neutral mass, Da	Identification	HMDB ID	Adducts	Identification level^2^	Food source of BFIs^3^
1.07	349.110	326.121	Blood group H disaccharide*	HMDB06590	+Na	1	Milk
1.14	530.207	529.200	Lewis A trisaccharide	HMDB06582	+H, +Na	1	Milk
1.02	219.047	196.058	Galactonic acid/gluconic acid*	HMDB00565/HMDB00625	+Na	3 (hydroxy acid)	Milk
1.07	162.076	161.068	Aminoadipic acid*	HMDB00510	+H, +2Na-H, +Na, +H-H_2_O	1	Cheese
0.99	176.103	175.095	Citrulline	HMDB00904	+H, +2Na-H, +Na	1	Cheese
1.18	219.134	218.127	Valyl-threonine	HMDB29137	+H, 2M+H	1	Cheese
5.08	263.139	262.131	Phenylalanyl-proline*	HMDB11177	+H	1	Cheese
6.55	206.081	205.074	Indolelactic acid*	HMDB00671	+H, +Na, +NH_4_, +2Na-H	1	Cheese
1.07	116.071	115.063	Proline	HMDB00162	+H, +Na, +2Na-H	1	Cheese

1 *n*  = 10 subjects. The isomers galactonic acid and gluconic acid could not be differentiated. *BFIs also detected in urine. Postprandial kinetics in serum and urine are detailed in Supplemental Figures 7–15. BFI, biomarker of food intake; HMDB ID, Human Metabolome Database Identification number; OPLS-DA, orthogonal projections to latent structures discriminant analysis.

2 Identification levels: 1, identified by comparison to a pure reference; 3, putatively characterized compound classes.

3 Selected as a BFI when VIP score >1.5 in 1-by-2 OPLS-DA analyses.

**Supplemental Figures 7–15** show the serum and urine postprandial kinetics of the 8 putative BFIs, and **Supplemental Tables 2** and **3** present the *P* values for the Kruskal-Wallis and the Conover-Iman tests at each time point.

### LeA and BGH after milk intake

**FIGURE 3:**
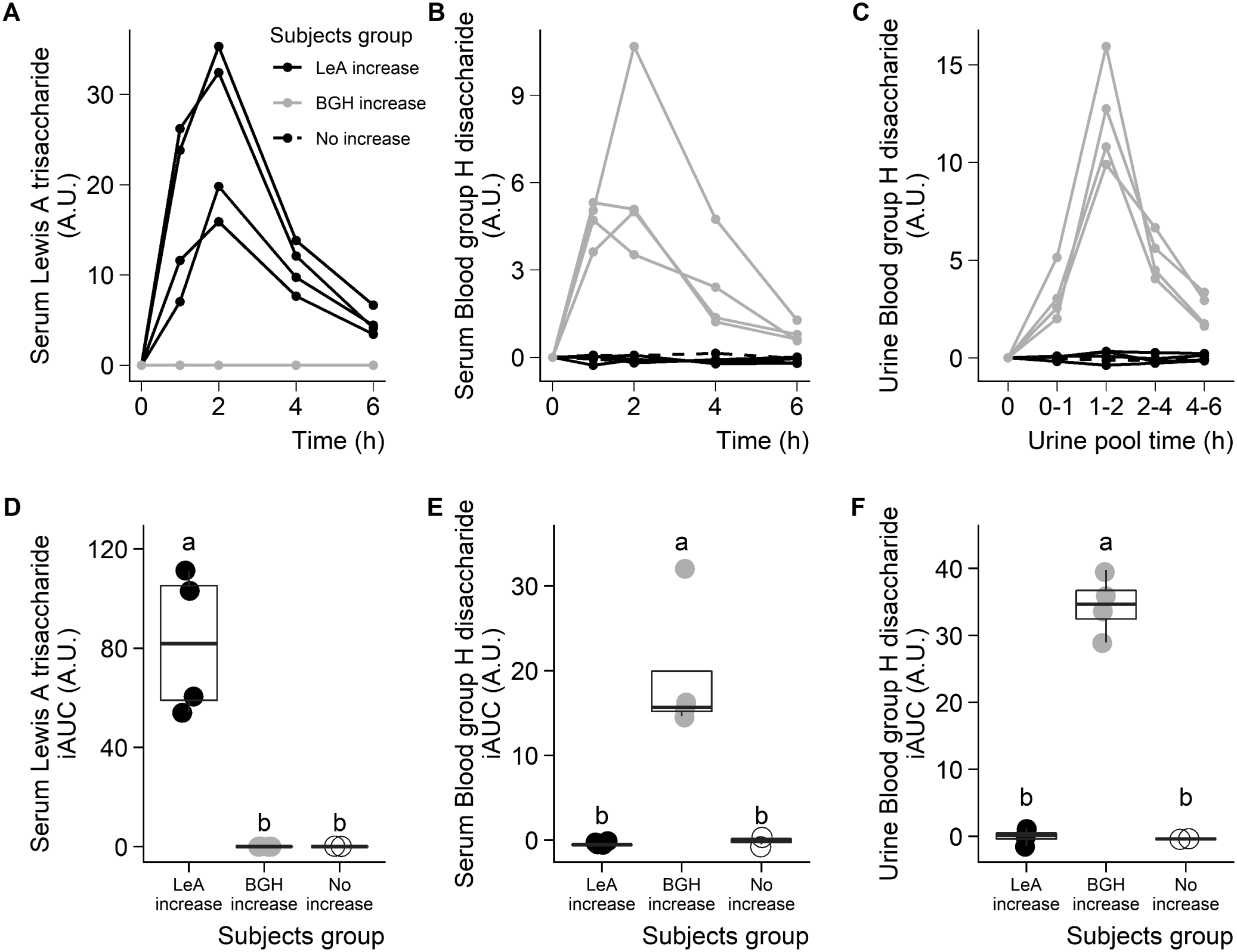
Interindividual variability in LeA and the BGH after milk intake in healthy adults. Serum LeA (A), serum BGH (B), and urine BGH (C) measured 6 h postprandially after milk intake in ten healthy subjects. Subjects were separated into three groups according to their postprandial response: LeA increase (*n* = 4), BGH increase (*n* = 4), and no increase (*n*  = 2). Comparison of postprandial responses of the three subject groups used iAUCs (D–F), IQRs plotted with medians. Means with different superscript letters (a, b, c) denote significant differences using a Kruskal-Wallis test completed by a Conover-Iman pairwise comparison test ( *P* < 0.05). A.U., arbitrary units; BGH, blood group H disaccharide; iAUC, incremental AUC; LeA, Lewis A trisaccharide.

LeA and BGH were identified as putative BFIs for milk. Both showed significant postprandial responses (serum nparLD: *P* = 1.6 × 10^−2^ and *P* = 1.9 × 10^−2^, respectively), although their kinetics indicated high interindividual variability (Supplemental Figures 7 and 8). Interestingly, individual kinetics revealed that this variability was due to the presence of three distinct groups of subjects (**Figure 3**): subjects showing a postprandial increase for LeA but not for BGH (LeA increase group), subjects showing a postprandial increase for BGH but not for LeA (BGH increase group), and subjects who did not show any increase for either of the two metabolites (No increase group). The postprandial increase in LeA and BGH reached a peak at ∼2 h, and returned to baseline after 6 h. In both cases, no difference was visible in 24-h fasting serum. Interestingly, the patterns observed in serum for BGH were confirmed in urine (Figure 3C, F) but not for LeA as it was not detected in urine. Significant differences were observed between the three groups of subjects when comparing serum or urine iAUCs using a Kruskal-Wallis test completed by a Conover-Iman pairwise comparison test (Figure 3D–F; **Supplemental Table 4**).

## Discussion

### General trends

Using untargeted metabolomics, we observed that the intake of milk, cheese, or soy leads to distinct postprandial serum metabolomes. Likely due to the plant origin of the test food, soy intake results in the most specific response, in accordance with the observations of Münger et al. (12) and Trimigno et al. (13). The results show that the majority of metabolites have a postprandial response that is not specific to the intake of a single food. In fact, we expect that the fraction of the postprandial metabolome responding specifically to the intake of a single food would further decrease as the number of control foods is increased, rendering the identification of specific BFIs difficult.

The postprandial phase after acute intake showed its efficiency to identify putative BFIs; however, their validity was not confirmed in fasting serum 24 h after ingestion, as previously observed (12). As the heatmap of kinetics suggests, the concentrations of many of the discriminant metabolites returned to baseline values 6 h after ingestion, in line with the very nature of nutrition, which is characterized by the repetition of multiple daily meals across the lifespan. The transient nature of the postprandial metabolome generally indicates that the identified metabolites can likely only be used as short-term BFIs or as markers of the quality of the metabolic response of the human organism to food intake. Nevertheless, the small sample size of the study and the single intake tested here may have prevented confirmation of significant differences in fasting serum (if any). Therefore, a validation of the postprandial BFIs in fasting samples from observational cohorts is essential and may be efficient as such studies provide larger sample sizes and samples taken after repeated intakes (14, 30, 31).

Heatmaps also reveal that metabolites discriminant for milk, cheese, or soy drink intake differ in their overall postprandial kinetics (early or late increase or decrease). Surprisingly, about one-third of the metabolites discriminant for milk intake decrease postprandially while increasing after cheese or soy intake. This observation suggests that milk intake downregulates endogenous metabolites and pathways. Conversely, most of metabolites discriminant for cheese intake increase postprandially and, therefore, are most likely absorbed exogenous compounds. Interestingly, the BFIs for cheese have a significant lower mass distribution than the BFIs for milk. A similar observation was made when comparing milk and yogurt intake (32). This shift in the postprandial mass distribution of discriminant metabolites could be indicative of a “predigestive” effect of fermentation on milk, resulting in the production and the absorption of specific low-molecular-weight compounds (e.g., SCFAs, oligonucleotides, or free amino acids).

### Biomarkers of cheese intake

While aminoadipic acid was discriminant for cheese intake, its use as a BFI is not recommended as increases were also observed after milk and soy intake. Aminoadipic acid is an intermediate in lysine metabolism (33), and a significant postprandial increase in serum lysine was observed after the intake of all three foods (data not shown). The higher concentrations of aminoadipic acid in serum after cheese intake could be attributed to higher amounts in cheese as it has been identified as a product of fermentation (34, 35). Nevertheless, higher concentrations of aminoadipic acid have also been reported after meat and fish intake (36) and after purple grumixama intake (37).

In contrast to aminoadipic acid, citrulline was a clear BFI for cheese, especially after 1 and 2 h. Citrulline can be produced from dietary arginine, proline, or glutamine by enterocytes (38), and consequently its concentration in blood is used by clinicians to evaluate gut integrity (39). Stanstrup et al. (22) reported higher serum citrulline concentrations at 2 h postprandially after whey protein intake compared with casein, gluten, or cod protein intake, suggesting greater arginine or proline uptake. Although we did not observe a significant difference in arginine concentrations (data not shown), we did see higher proline concentrations after the intake of cheese. The production of citrulline from arginine by lactic acid bacteria has been described during soy, cabbage, or milk fermentation (40–42). It is thus likely that cheese had higher citrulline contents than milk or soy. The use of this molecule as a BFI for cheese has not been reported and needs to be further explored.

Two dipeptides with level 1 identification showed a clear increase after cheese intake. “Predigestion” of milk proteins by lactic acid bacteria during fermentation might explain the higher concentrations of peptides in serum after the ingestion of fermented dairy products compared with after milk intake (43). VT and FP are both present in milk protein sequences: β-lactoglobulin (VT), serum albumin (VT and FP), κ-casein (VT), ɑ-S2-casein (FP), and β-casein (FP) (44). Given that any dipeptide sequence should appear randomly every 400 amino acids along proteomes, the specificity of the two dipeptides as BFIs for cheese is questionable. Their relevance as a BFI has therefore to be confirmed since they are likely to be found in other dietary proteins. However, the frequency of valine, threonine, phenylalanine, and proline in major dairy proteins is higher compared with that in nondairy proteins (45), suggesting that the presence in serum or urine of these dipeptides, possibly in combination with others, might be useful biomarkers to differentiate nonfermented from fermented dairy consumption.

The release of free amino acids during cheese ripening has been well described (46) and would directly explain the higher concentration in serum of free proline after cheese intake [also observed using NMR (13) and GC-MS (12)]. It could also, indirectly, explain the increase in serum ILA observed in our study as ILA is an end-product of tryptophan metabolism via the indole pathway. Free tryptophan might be metabolized into ILA either by lactic acid bacteria during cheese ripening (32, 47) or by the host microbiota as intestinal *Lactobacilli* use tryptophan as a source of energy in high tryptophan conditions, notably through the indole pathway (48, 49). A similar increase in postprandial serum ILA has been described after yogurt intake compared with non–fermented milk intake, with a persisting trend in fasting serum after 2 wk of daily consumption (32). These observations qualify ILA (and potentially other indole derivatives) as candidate BFIs for fermented dairy consumption.

### Biomarkers of milk intake

The presence of galactonic acid in serum samples is likely due to the oxidation of serum galactose, which is known to increase postprandially following the ingestion of a high quantity of milk (20, 21). Galactonic acid was previously detected in postprandial serum and urine samples from the same study using GC-MS (12, 13), confirming its potential use as a biomarker of milk intake, as proposed by Vionnet et al. (21) in postprandial samples and Playdon et al. (50) in fasting serum samples. While measurement using LC-MS could not differentiate galactonic acid from its isomer gluconic acid, the above-mentioned literature suggests that the biomarker would indeed be galactonic acid.

### LeA and BGH as biomarkers of milk intake subject to interindividual variability

**FIGURE 4:**
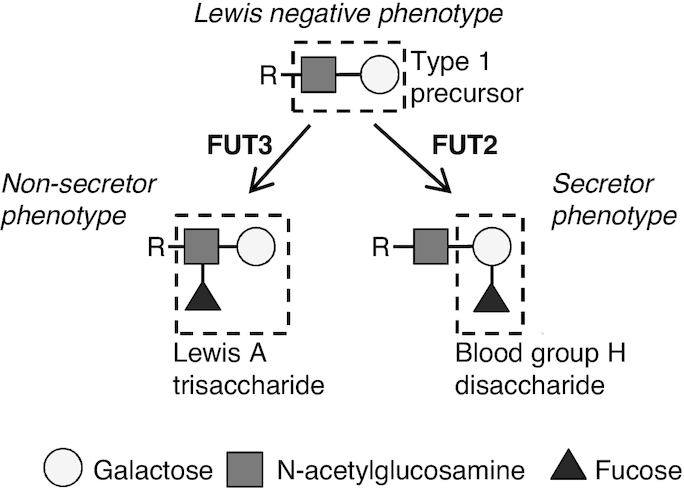
Schematic diagram for the formation of BGH or LeA motives on a type 1 precursor chain, in secretor and nonsecretor subjects. BGH, blood group H disaccharide; FUT2, fucosyl transferase 2; FUT3, fucosyl transferase 3; LeA, Lewis A trisaccharide.

The presence of LeA and BGH in serum is particularly interesting as it demonstrates, for the first time, their appearance in adults after the ingestion of bovine milk. LeA and BGH are two fucosylated oligosaccharides composed of galactose-fucose-N-acetylglucosamine and fucose-galactose, respectively (**Figure 4**). Contrary to human milk oligosaccharides, bovine milk oligosaccharides are not fucosylated (51, 52), or are in a very limited proportion (<1%) (53); the two compounds might therefore be of endogenous origin. In humans, fucosylation of carbohydrates occurs in the epithelial lining of the pulmonary, urinary, reproductive, and most importantly, gastrointestinal tract. The resulting fucosylated compounds are then found at the tissue surface, in the respective mucosal secretions, as well as in plasma (54, 55). Intestinal fucosylation takes place in the brush-border enterocytes and mainly relies on expression of the fucosyltransferase (*FUT*) 2 and 3 genes (*FUT2*, *FUT3*), which control the addition of a fucose molecule, respectively, on the galactose or the N-acetylglucosamine of a type 1 precursor chain (Figure 4) (55). FUT2 and FUT3 enzymes act in competition, with FUT2 having greater activity. This antigenic pattern consisting of a fucosylated type 1 precursor is at the basis of the Lewis antigen system. In humans, three main Lewis phenotypes have been described due to polymorphisms on the FUT2 and FUT3 genes: secretor status (FUT2 active, resulting in fucosylated galactose, ∼72% in Caucasians), nonsecretor status (FUT2 inactive, FUT3 active, resulting in fucosylated N-acetylglucosamine, ∼22% in Caucasians), and Lewis negative (FUT2 and FUT3 both inactive, resulting in no fucosylation of the precursor chain, ∼6% in Caucasians) (55). Polymorphism in *FUT* genes could therefore explain the interindividual variability observed in our study for the BGH and LeA response after milk intake and the presence of three clear groups of subjects: postprandial increase in BGH (fucosylated galactose), postprandial increase in LeA (fucosylated N-acetylglucosamine), or no increase.

The biological benefits of milk oligosaccharides and fucosylation have been extensively investigated, in particular regarding the benefits of human breast milk for newborns (56–58). Fucosylated oligosaccharides at the intestinal surface are used as targets by various pathogenic bacteria and viruses to adhere to the epithelium (59–61). The high concentrations of free LeA in human milk might thus act as an analog to inhibit the binding of pathogens in newborns (62). The link between Lewis antigen phenotype and susceptibility to various gut infections has been demonstrated in children and adults. In particular, minor alleles of *FUT3* polymorphisms appear to increase susceptibility to ulcerative colitis (63), whereas minor alleles of *FUT2* polymorphism are associated with increased susceptibility to celiac disease (64), as well as increased risks of *Escherichia coli* (65) and *Staphylococcus aureus* (66) infections. On the other hand, the same *FUT2* polymorphisms are associated with resistance to norovirus (67–69) and rotavirus (70) infections and reduced risk of diarrhea (71). Furthermore, the secretor/nonsecretor status has been associated with susceptibility to various diseases such as type 1 diabetes (72), asthma (73, 74), and cardiovascular diseases (75). Finally, intestinal fucosylated glycan structures can be used as a substrate by specific colonic bacteria such as *Bifidobacterium* species or *Ruminococcus*, *Clostridium*, and *Akkermansia* genera (76, 77). Consequently, the secretor/nonsecretor status has a key influence on the microbiota composition, in particular on the *Bifidobacteria* population (78, 79), an effect that could be modulated by diet (80).

The limited sample size of our study did not allow for powerful statistical comparison of the three subject groups observed here; however, the clear postprandial kinetics with five consecutive time points and the dichotomic behavior of the two metabolites, which is in line with current knowledge on the biochemical pathways and genetic polymorphisms leading to the production of Lewis A antigens, underline the plausibility of our findings and encourage further investigations in independent larger studies.

In conclusion, by extending the analysis of the serum and urine metabolomes using LC-MS, we could identify new candidate BFIs for milk, cheese, and soy. Among the identified BFIs, only two (proline and galactonic/gluconic acid) were previously identified using GC-MS and NMR, highlighting the relevance of multiplatform metabolomics. However, the validation of BFIs is a complex process and requires testing their plausibility, dose–response, time–response, robustness, reliability, stability, analytical performance, and interlaboratory reproducibility (14). The presence of LeA and BGH in serum of adults after milk intake is of interest as such metabolites have mainly been discussed in the context of maternal milk and newborns. However, the link between the presence of LeA and BGH and the subjects’ secretor or nonsecretor status still has to be confirmed. Nevertheless, considering the extensive effects on health related to the presence or absence of such antigenic motifs, it would be of interest to investigate if secretors benefit from drinking milk differently from nonsecretor subjects.

## Supplementary Material

nxaa029_Supplemental_Files

## Notes

The study was funded by the Swiss National Science Foundation (40HD40_160618) in the frame of the national research program “Healthy Nutrition and Sustainable Food Protection” (NRP69) and the European Healthy Diet for a Healthy Life Joint Programming Initiative “The Food Biomarkers Alliance” (FoodBAll).

Author disclosures: The authors report no conflicts of interest.

Supplemental Tables 1–4, Supplemental Figures 1–15, and Supplemental Methods are available from the “Supplementary data” link the online posting of the article and from the same link in the online table of contents at https://academic.oup.com/jn/.

Abbreviations used: BFI, biomarker of food intake; BGH, blood group H disaccharide; CV-ANOVA, ANOVA of the cross-validated residuals; FoodBAll, Food Biomarkers Alliance; FP, phenylalanyl-proline; *FUT*, fucosyltransferase; iAUC, incremental AUC; ILA, indole-3-lactic acid; LeA, Lewis A trisaccharide; nparLD, nonparametric analysis of longitudinal data; OPLS-DA, orthogonal projections to latent structures discriminant analysis; PLS-DA, partial least-square discriminant analysis; VIP, variable importance in projection for the predictive component; VT, valyl-threonine.
