# Supplementary material for: Identification of Milk and Cheese Intake Biomarkers in Healthy Adults Reveals High Interindividual Variability of Lewis System–Related Oligosaccharides
Source: J Nutr. 2020 Mar 4;150(5):1058–67. doi: 10.1093/jn/nxaa029 (PMC7198293; doi:10.1093/jn/nxaa029)
Supplement: nxaa029_Supplemental_Files [file nxaa029_supplemental_files.zip › G_Pimentel_JNutr_Supplemental_Figures_Revised_21_01_20.pdf]

Identification of milk and cheese intake biomarkers in healthy adults reveals high inter-individual variability of Lewis system related oligosaccharides.

Grégory Pimentel

Online Supplementary Material

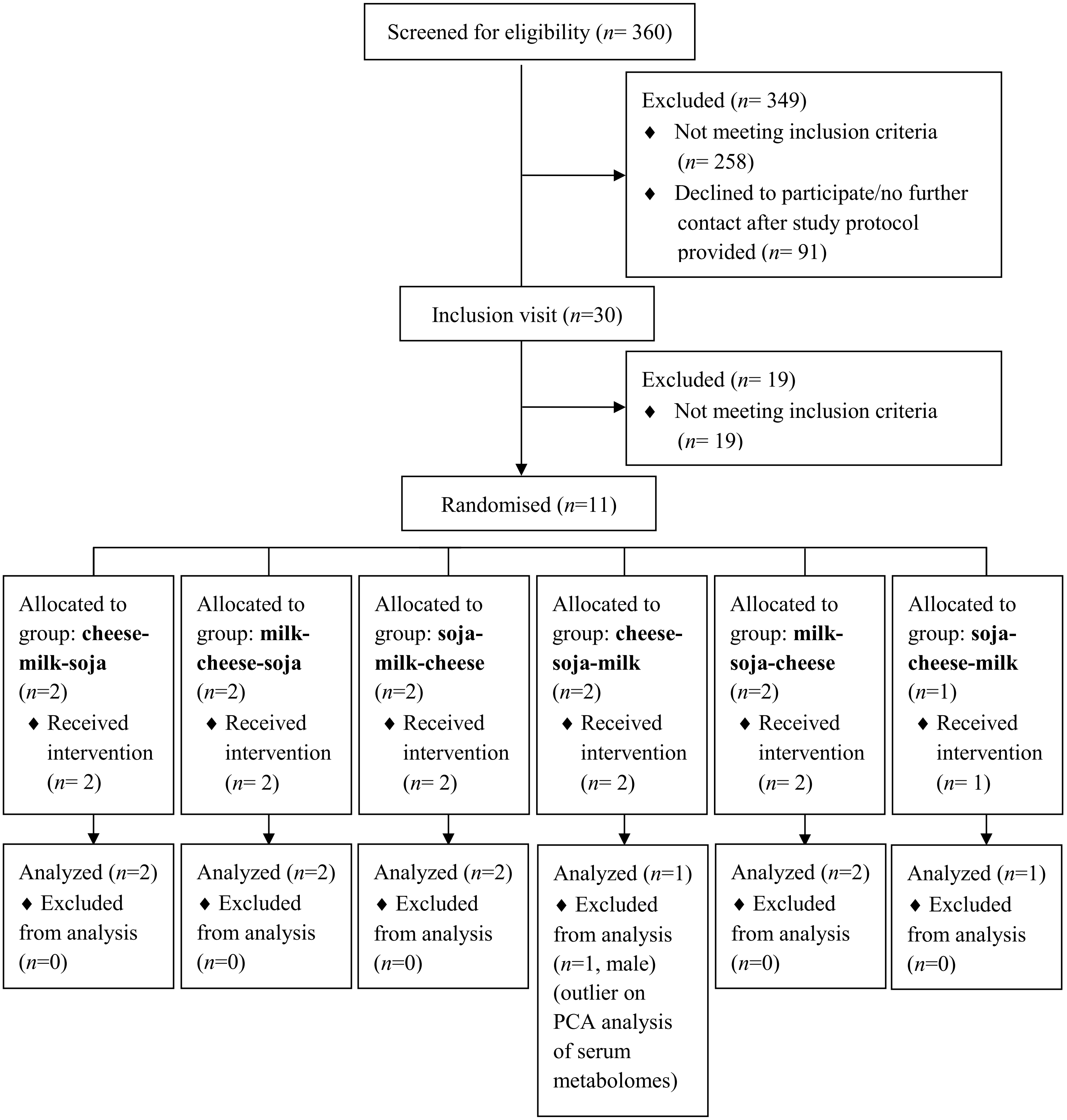

SUPPLEMENTAL FIGURE 1 Flow chart of the FoodBAll dairy study (adapted with permission from Burton *et al.* (1))**.**

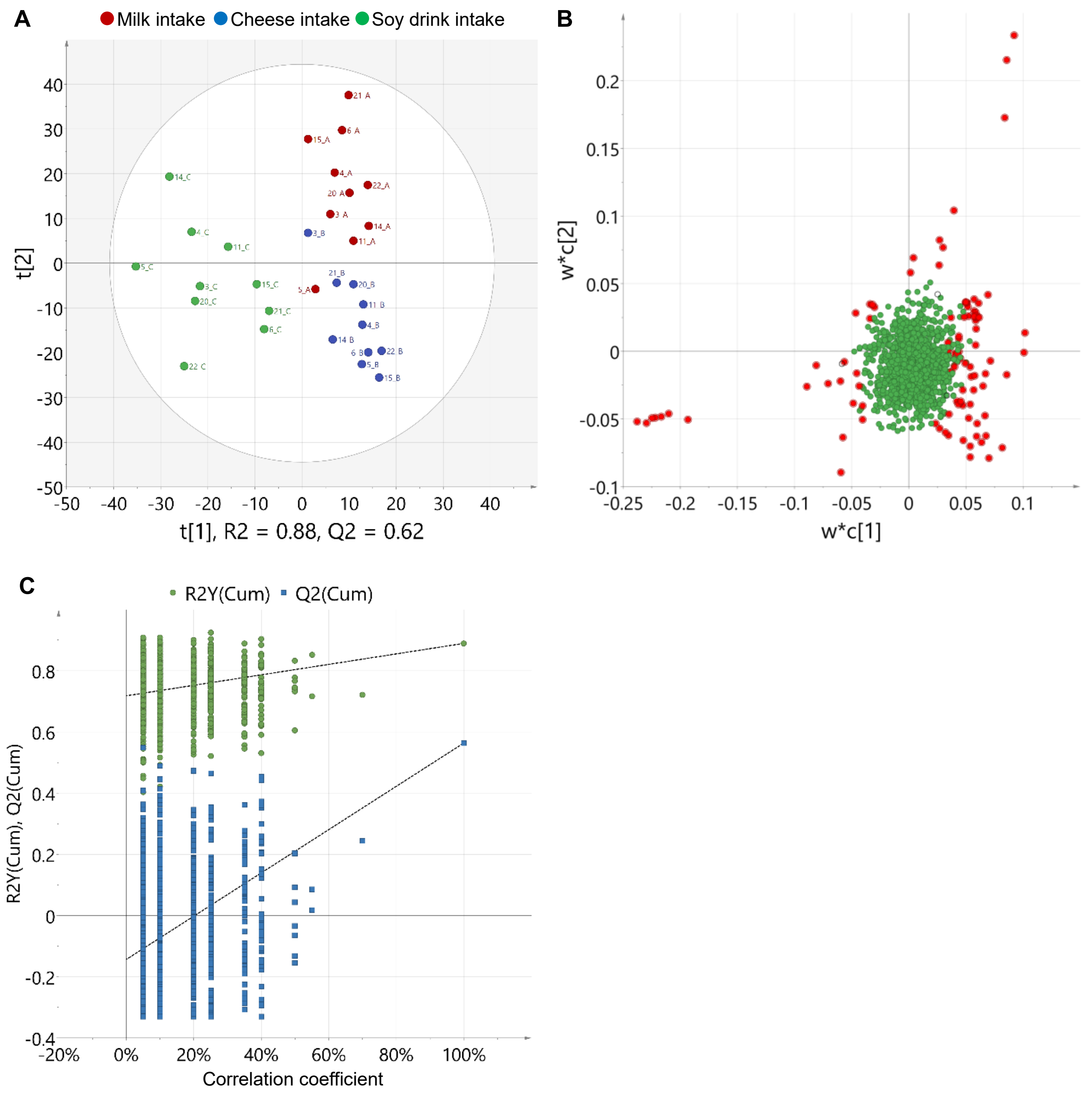

SUPPLEMENTAL FIGUR E 2 Partial least squares discriminant analysis derived from postprandial serum taken after milk, cheese, or soy drink intake in healthy subjects. Three components, R2Y (cum) = 0.88, Q2 = 0.62. (A) PLS-DA score plot of samples taken after the ingestion of milk (A, red), cheese (B, blue) or soy drink (C, green) (n = 10 participants). (B) Loading plot of the 1639 serum metabolites with a significant postprandial response after milk and/or cheese and/or soy intake, discriminant metabolites having a VIP score > 1.5 are in red. (C) Permutation test with 999 permutations between original and permuted Y-vector using one component. The defined model was validated by higher original values of R2Y and Q2 than values obtained after permutations.

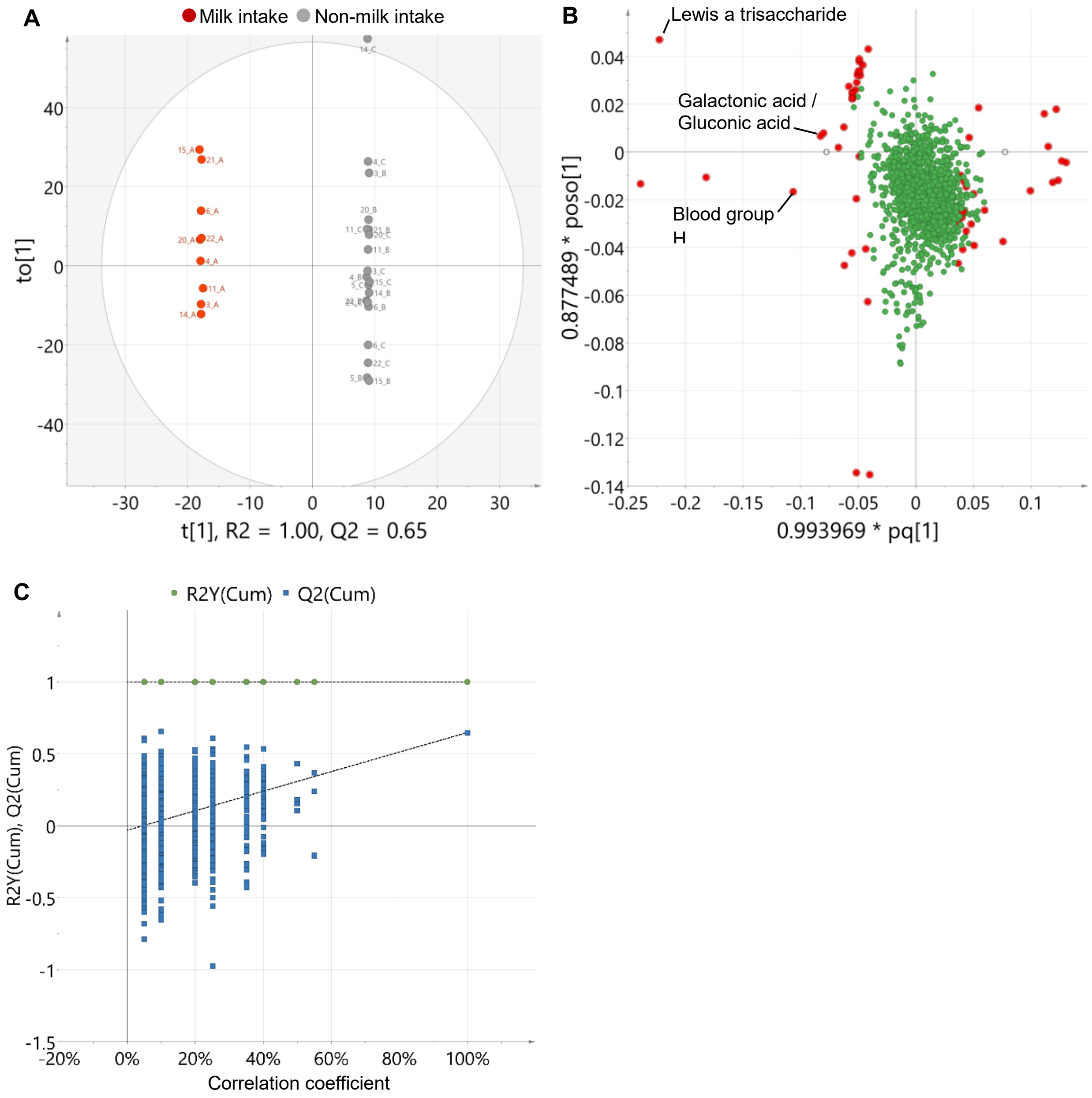

SUPPLEMENTAL FIGURE 3 Orthogonal partial least squares discriminant analysis derived from postprandial serum taken after milk, cheese, or soy drink intake in healthy subjects. Number of components: 1+6+0, R2Y (cum) = 1.00, Q2 = 0.65. (A) OPLS-DA score plot of samples taken after the ingestion of milk (red) or cheese/soy drink (grey) (*n* = 10 participants). (B) Loading plot of the 1639 serum metabolites with a significant postprandial response after milk and/or cheese and/or soy intake, discriminant metabolites having a VIP score > 1.5 are in red, metabolites identified at level 1 are indicated. (C) Permutation test with 999 permutations between original and permuted Y-vector using one component. The defined model was validated by higher original values of R2Y and Q2 than values obtained after permutations.

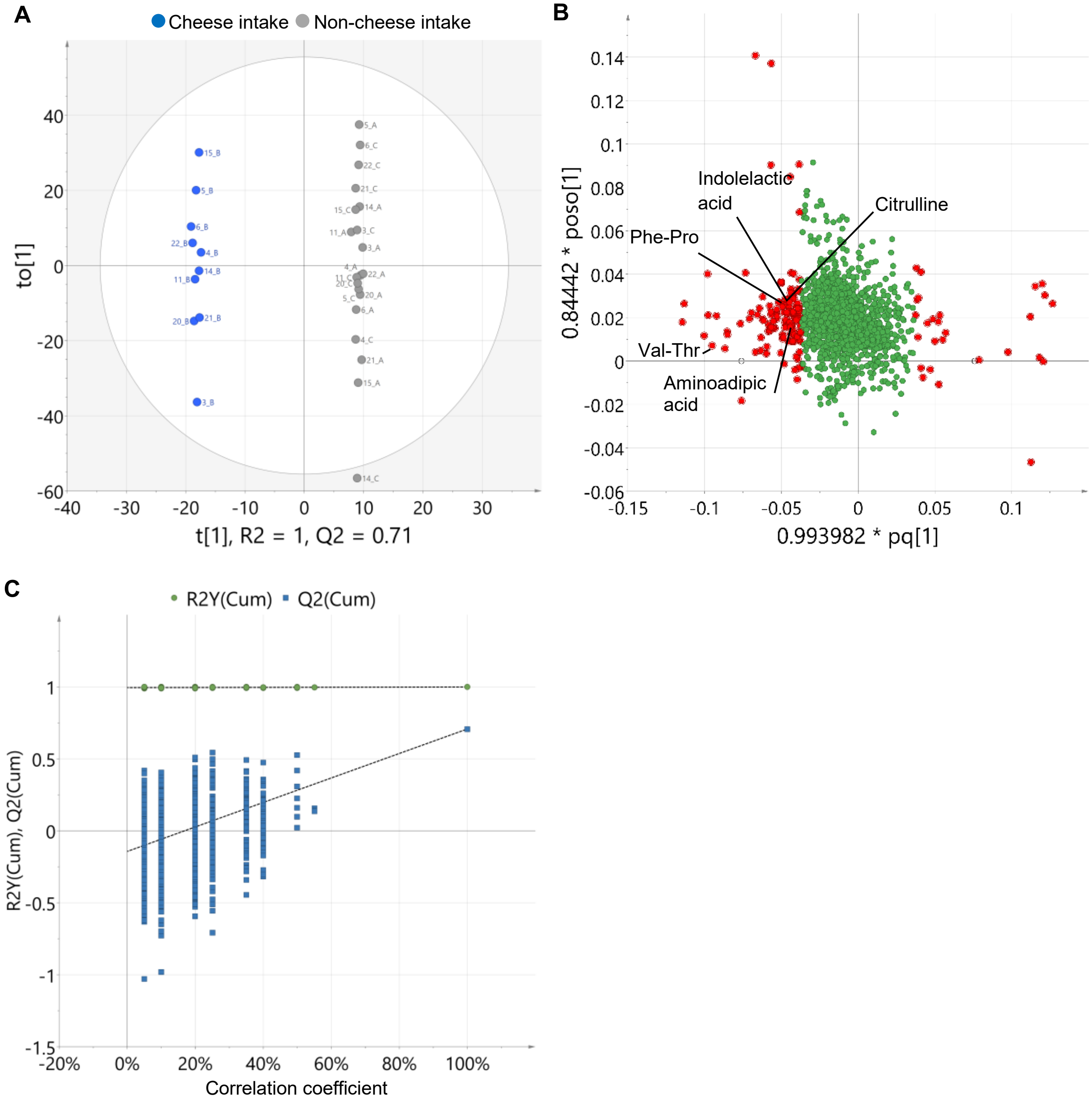

SUPPLEMENTAL FIGURE 4 Orthogonal partial least squares discriminant analysis derived from postprandial serum taken after milk, cheese, or soy drink intake in healthy subjects. Number of components: 1+4+0, R2Y (cum) = 1.00, Q2 = 0.71. (A) OPLS-DA score plot of samples taken after the ingestion of cheese (blue) or milk/soy drink (grey) (*n* = 10 participants). (B) Loading plot of the 1639 serum metabolites with a significant postprandial response after milk and/or cheese and/or soy intake, discriminant metabolites having a VIP score > 1.5 are in red, metabolites identified at level 1 are indicated. (C) Permutation test with 999 permutations between original and permuted Y-vector using one component. The defined model was validated by higher original values of R2Y and Q2 than values obtained after permutations.

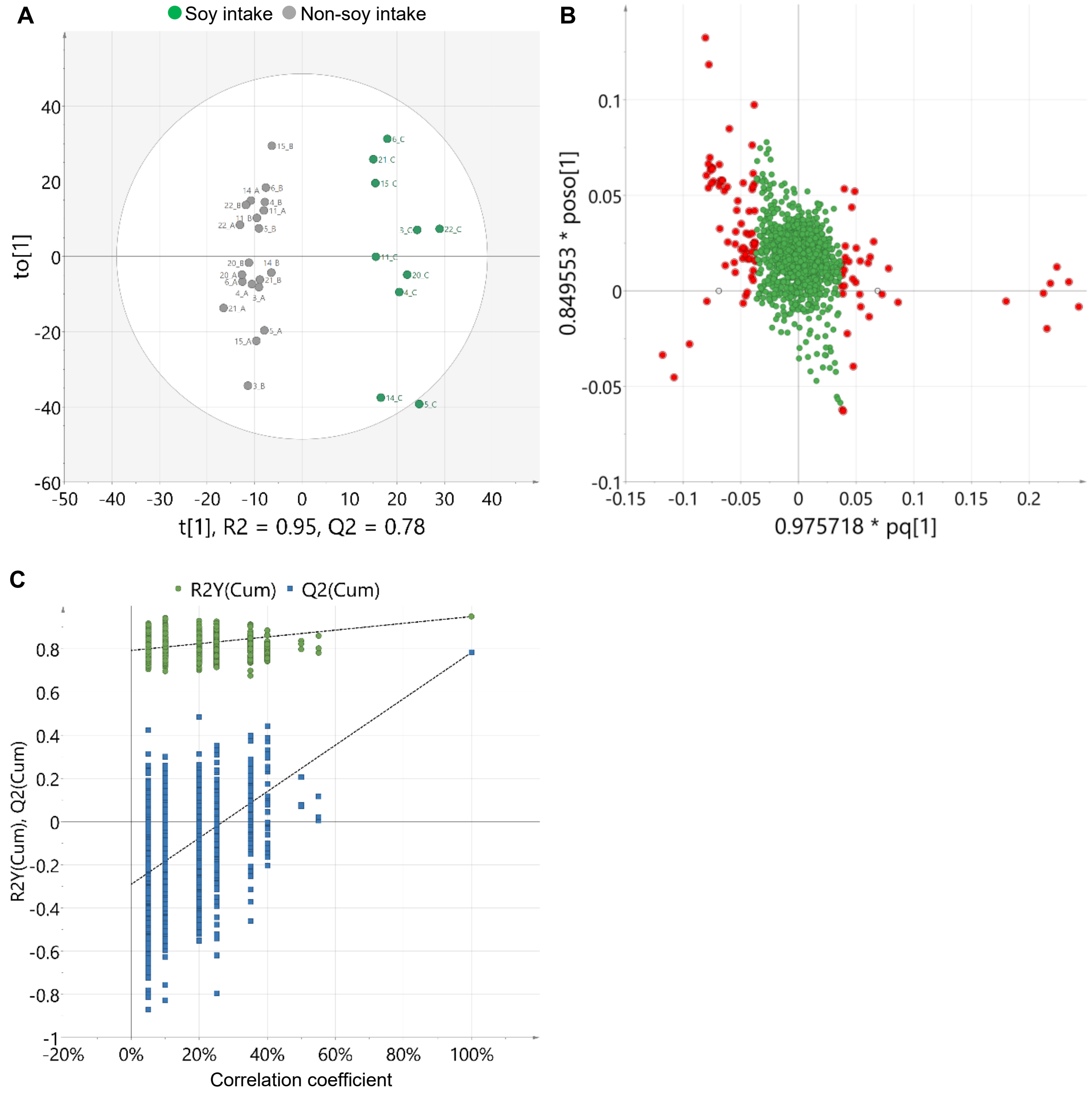

SUPPLEMENTAL FIGURE 5 Orthogonal partial least squares discriminant analysis derived from postprandial serum taken after milk, cheese, or soy drink intake in healthy subjects. Number of components: 1+1+0, R2Y (cum) = 0.95, Q2 = 0.78. (A) OPLS-DA score plot of samples taken after the ingestion of soy (green) or milk/cheese drink (grey) (n = 10 participants). (B) Loading plot of the 1639 serum metabolites with a significant postprandial response after milk and/or cheese and/or soy intake, discriminant metabolites having a VIP score > 1.5 are in red. (C) Permutation test with 999 permutations between original and permuted Y-vector using one component. The defined model was validated by higher original values of R2Y and Q2 than values obtained after permutations.

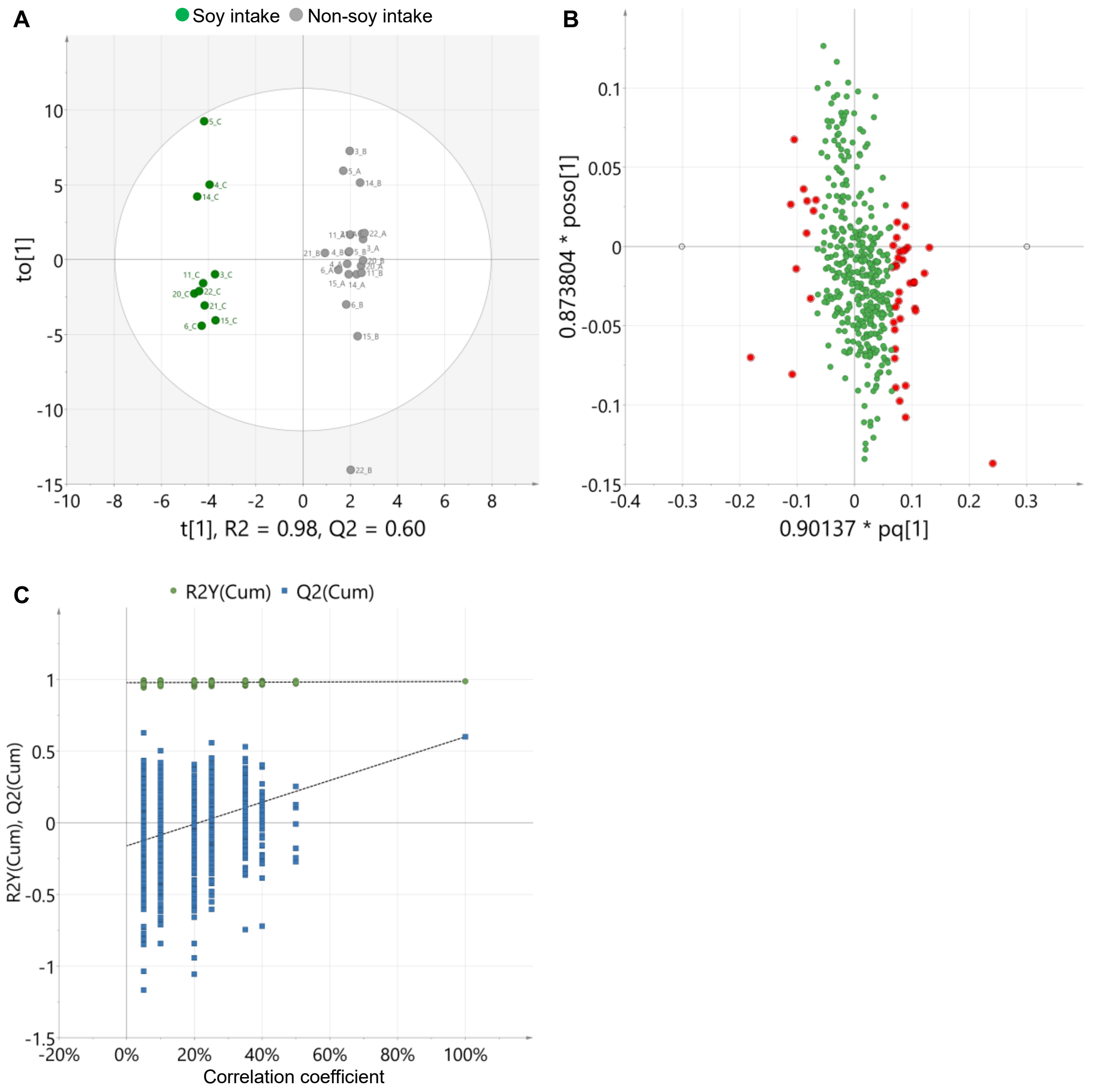

SUPPLEMENTAL FIGURE 6 Orthogonal partial least squares discriminant analysis derived from fasting serum taken 24 h after milk, cheese, or soy drink intake in healthy subjects. Number of components: 1+4+0, R2Y (cum) = 0.95, Q2 = 0.60. (A) OPLS-DA score plot of samples taken after the ingestion of soy (green) or milk/cheese drink (grey) (*n* = 10 participants). (B) Loading plot of the 430 serum metabolites with a significant change in fasting serum after milk and/or cheese and/or soy intake, discriminant metabolites having a VIP score > 1.5 are in red. (C) Permutation test with 999 permutations between original and permuted Y-vector using one component. The defined model was validated by higher original values of R2Y and Q2 than values obtained after permutations.

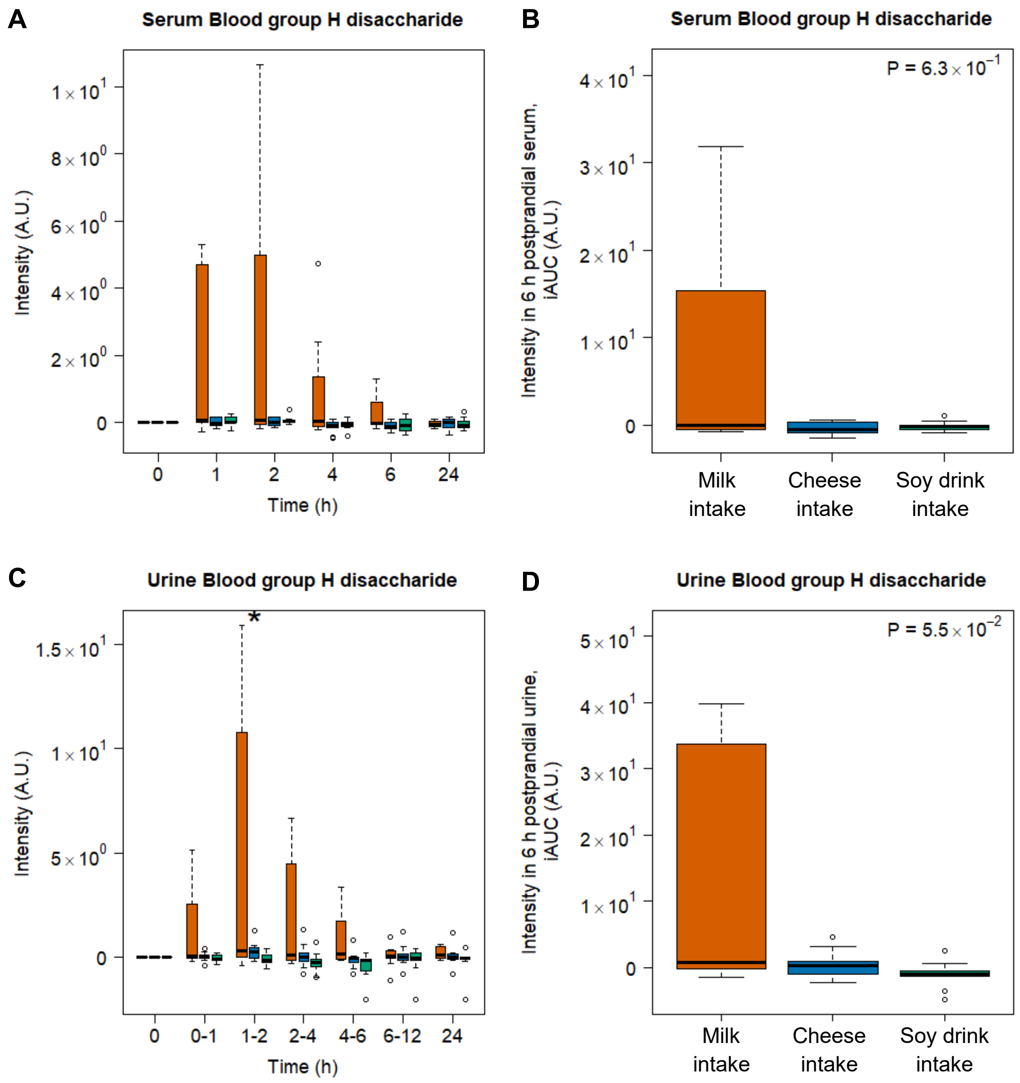

SUPPLEMENTAL FIGURE 7 Blood group H disaccharide after milk, cheese or soy drink intake in serum and urine of healthy subjects. Interquartile range plotted with minimum, maximum, median (—) and outliers (◦), *n* = 10 participants. Left panel shows the intensity measured by LC-MS in serum or urine, 6 h postprandially and fasting 24 h after the ingestion of milk (orange), cheese (blue) or a soy drink (green). *, significant food effect using the Kruskall-Wallis test ($P < 0.05$, adjusted for multiple testing). Right panel shows the 6 h iAUC in serum or urine after milk, cheese or soy drink intake. *P* values adjusted for multiple testing using the Kruskall-Wallis test are indicated. Detailed *P* values for the Kruskal-Wallis test for each time points are given in Supplemental Tables 2 and 3.

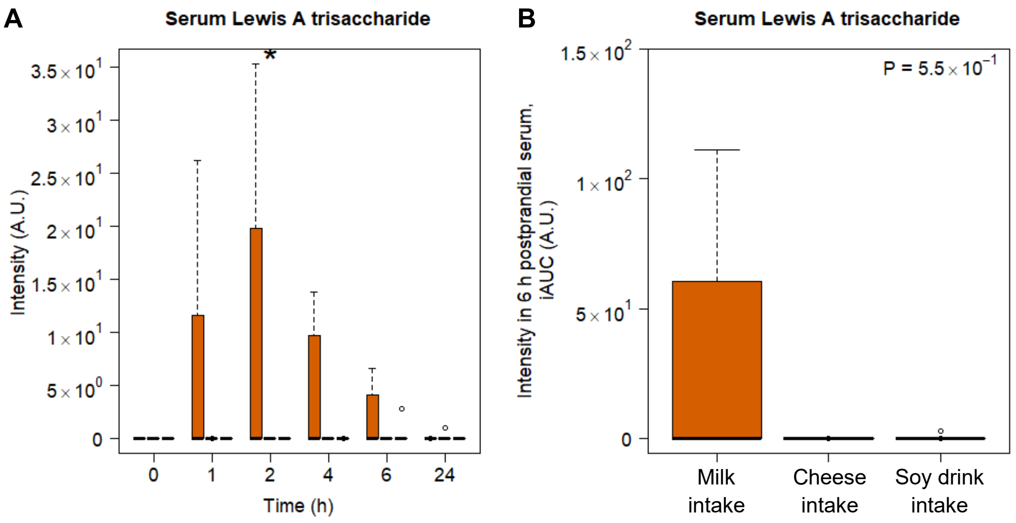

SUPPLEMENTAL FIGURE 8 Lewis A trisaccharide after milk, cheese or soy drink intake in serum of healthy subjects. Interquartile range plotted with minimum, maximum, median (—) and outliers (◦), $n = 10$ participants. (A) Intensity measured by LC-MS in serum, 6 h postprandially and fasting 24 h after the ingestion of milk (orange), cheese (blue) or a soy drink (green). *, significant food effect using the Kruskall-Wallis test ($P < 0.05$, adjusted for multiple testing). (B) 6 h iAUC in serum after milk, cheese or soy drink intake. P values adjusted for multiple testing using the Kruskall-Wallis test are indicated. Detailed P values for the Kruskal-Wallis test for each time points are given in Supplemental Tables 2.

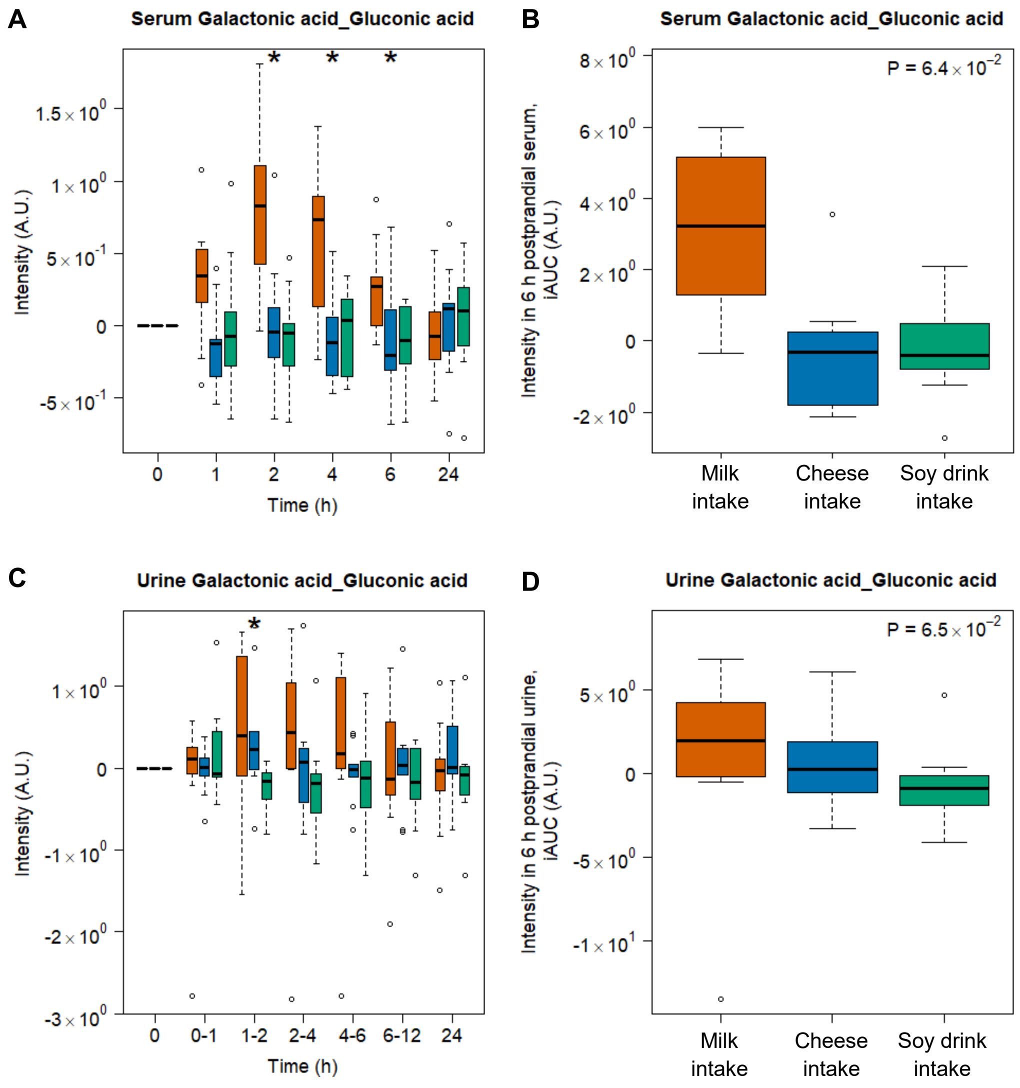

SUPPLEMENTAL FIGURE 9 Galactonic acid / Gluconic acid after milk, cheese or soy drink intake in serum and urine of healthy subjects. Interquartile range plotted with minimum, maximum, median (—) and outliers (◦), *n* = 10 participants. Left panel shows the intensity measured by LC-MS in serum or urine, 6 h postprandially and fasting 24 h after the ingestion of milk (orange), cheese (blue) or a soy drink (green). *, significant food effect using the Kruskall-Wallis test ($P < 0.05$, adjusted for multiple testing). Right panel shows the 6 h iAUC in serum or urine after milk, cheese or soy drink intake. *P* values adjusted for multiple testing using the Kruskall-Wallis test are indicated. Detailed *P* values for the Kruskal-Wallis test for each time points are given in Supplemental Tables 2 and 3.

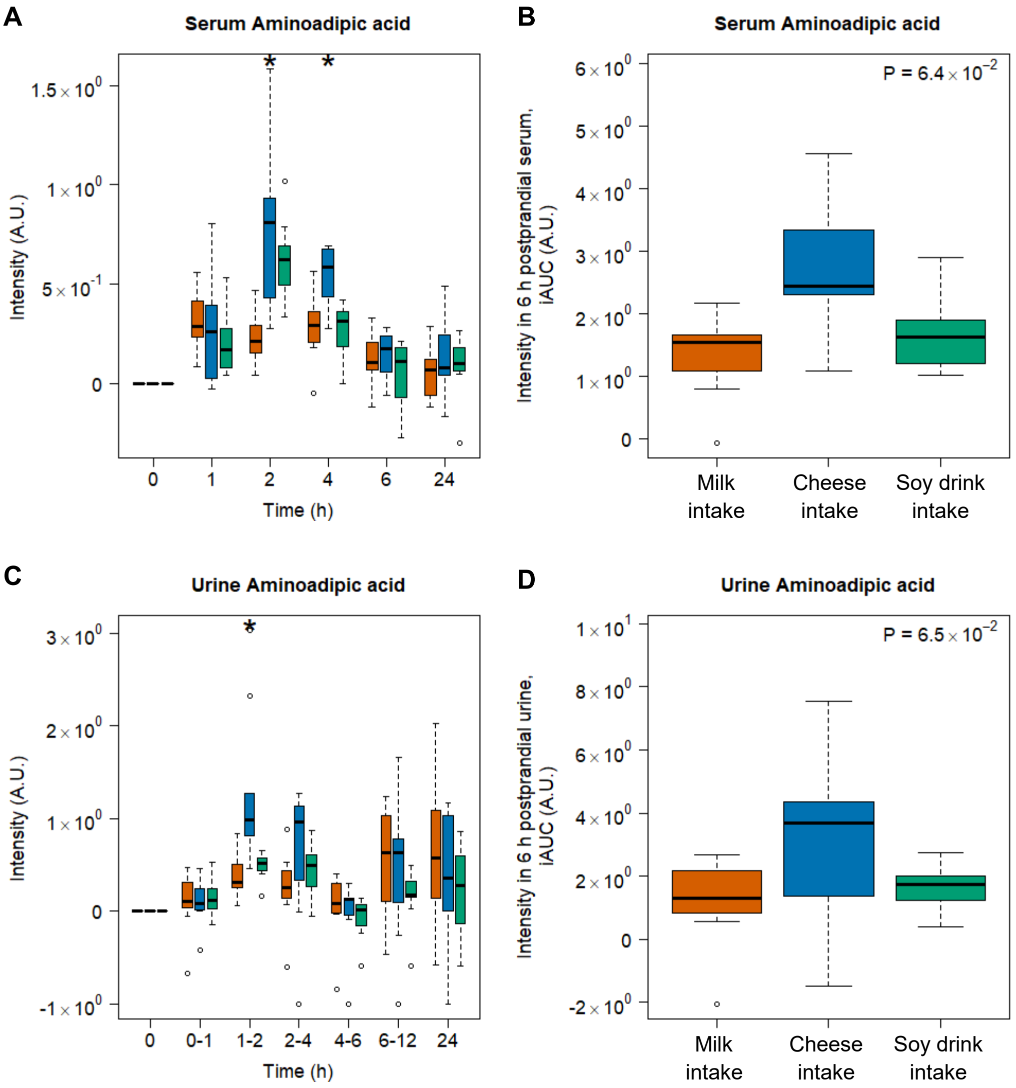

SUPPLEMENTAL FIGURE 10 Aminoadipic acid after milk, cheese or soy drink intake in serum and urine of healthy subjects. Interquartile range plotted with minimum, maximum, median (—) and outliers (◦), *n* = 10 participants. Left panel shows the intensity measured by LC-MS in serum or urine, 6 h postprandially and fasting 24 h after the ingestion of milk (orange), cheese (blue) or a soy drink (green). *, significant food effect using the Kruskall-Wallis test ($P < 0.05$, adjusted for multiple testing). Right panel shows the 6 h iAUC in serum or urine after milk, cheese or soy drink intake. *P* values adjusted for multiple testing using the Kruskall-Wallis test are indicated. Detailed *P* values for the Kruskal-Wallis test for each time points are given in Supplemental Tables 2 and 3.

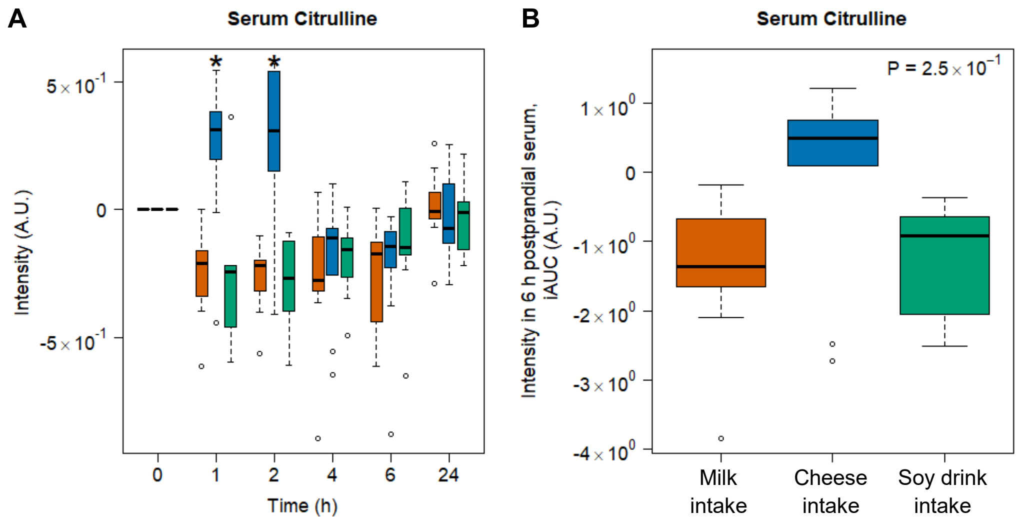

SUPPLEMENTAL FIGURE 11 Citrulline after milk, cheese or soy drink intake in serum of healthy subjects. Interquartile range plotted with minimum, maximum, median (—) and outliers (◦), n = 10 participants. (A) Intensity measured by LC-MS in serum, 6 h postprandially and fasting 24 h after the ingestion of milk (orange), cheese (blue) or a soy drink (green). *, significant food effect using the Kruskall-Wallis test (P < 0.05, adjusted for multiple testing). (B) 6 h iAUC in serum after milk, cheese or soy drink intake. P values adjusted for multiple testing using the Kruskall-Wallis test are indicated. Detailed P values for the Kruskal-Wallis test for each time points are given in Supplemental Tables 2.

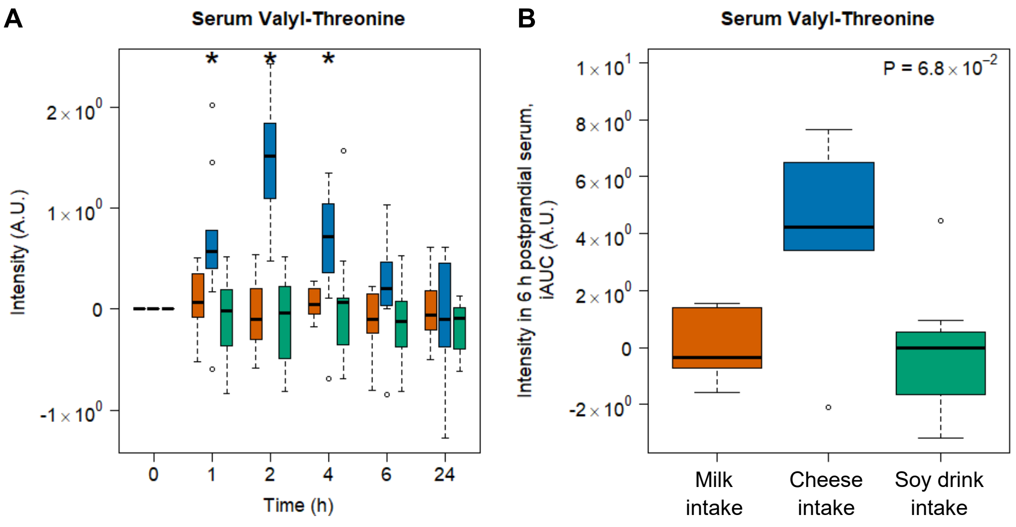

SUPPLEMENTAL FIGURE 12 Valyl-Threonine after milk, cheese or soy drink intake in serum of healthy subjects. Interquartile range plotted with minimum, maximum, median (—) and outliers (◦), *n* = 10 participants. (A) Intensity measured by LC-MS in serum, 6 h postprandially and fasting 24 h after the ingestion of milk (orange), cheese (blue) or a soy drink (green). *, significant food effect using the Kruskall-Wallis test ($P < 0.05$, adjusted for multiple testing). (B) 6 h iAUC in serum after milk, cheese or soy drink intake. *P* values adjusted for multiple testing using the Kruskall-Wallis test are indicated. Detailed *P* values for the Kruskal-Wallis test for each time points are given in Supplemental Tables 2.

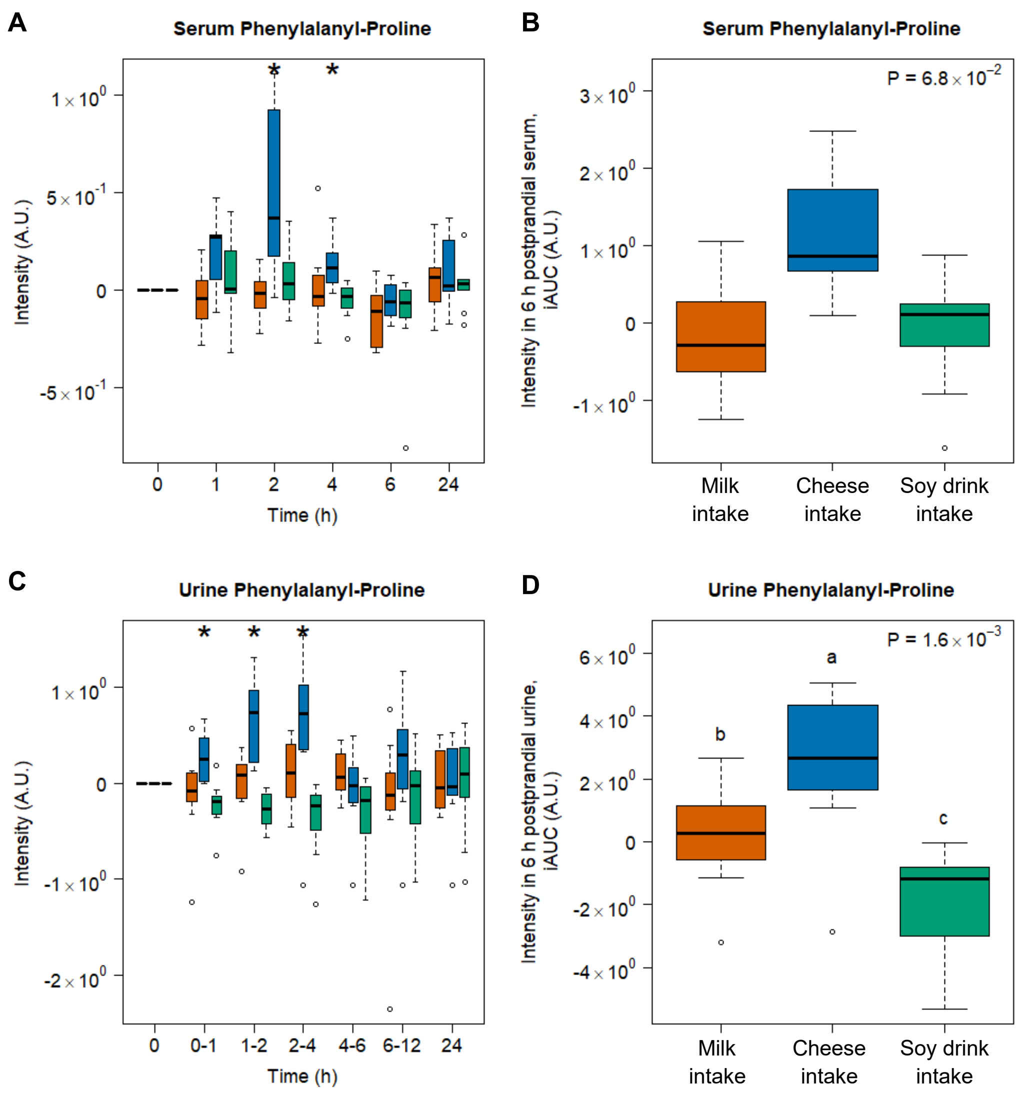

SUPPLEMENTAL FIGURE 13 Phenylalanyl-Proline after milk, cheese or soy drink intake in serum and urine of healthy subjects. Interquartile range plotted with minimum, maximum, median (—) and outliers (◦), n = 10 participants. Left panel shows the intensity measured by LC-MS in serum or urine, 6 h postprandially and fasting 24 h after the ingestion of milk (orange), cheese (blue) or a soy drink (green). *, significant food effect using the Kruskall-Wallis test (P < 0.05, adjusted for multiple testing). Right panel shows the 6 h iAUC in serum or urine after milk, cheese or soy drink intake. P values adjusted for multiple testing using the Kruskall-Wallis test are indicated. If the Kruskal-Wallis test was significant (P < 0.05), pairwise comparisons were conducted using a Conover-Inman test. Different letters (a, b, c) denote significant differences based on a P value < 0.05. Detailed P values for the Kruskal-Wallis and Conover-Inman tests and for each time points are given in Supplemental Tables 2 and 3.

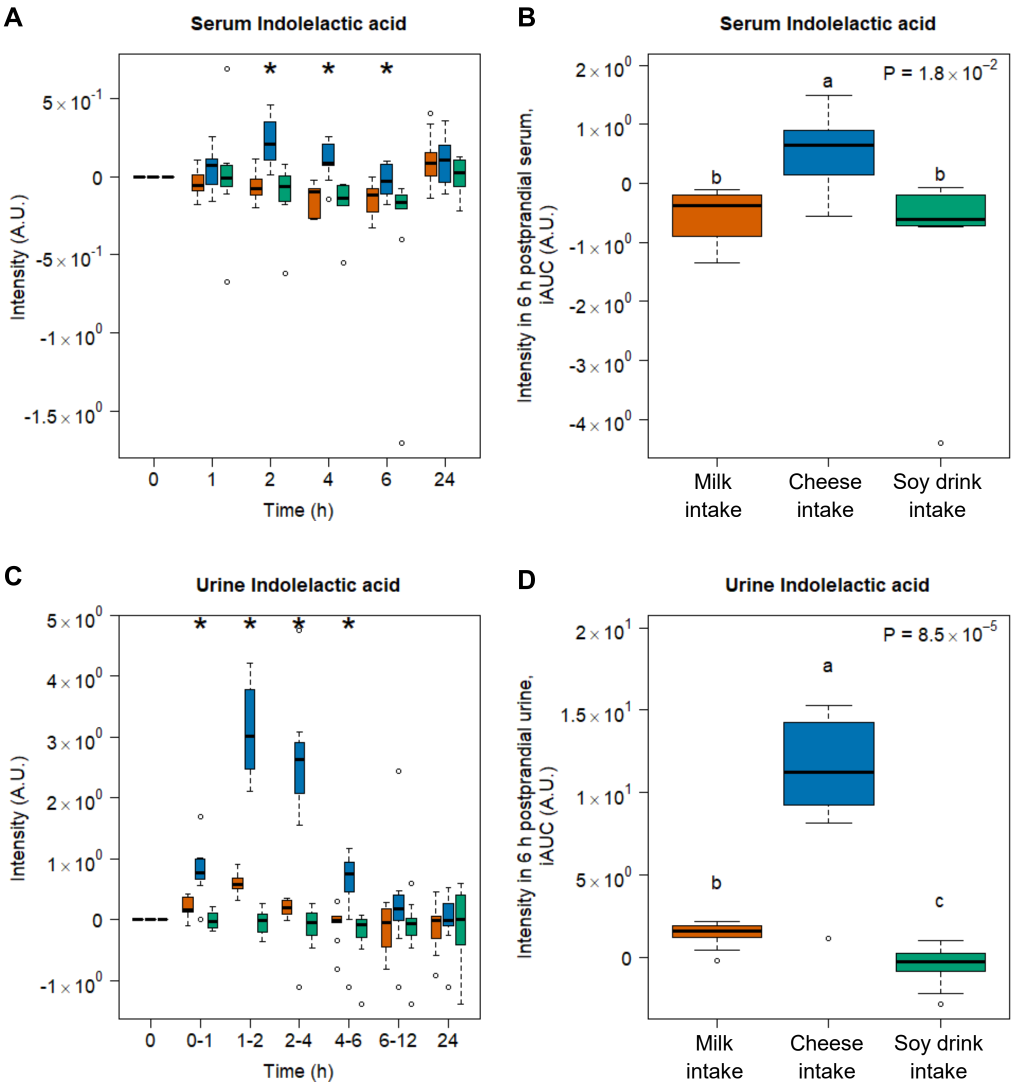

SUPPLEMENTAL FIGURE 14 Indolelactic acid after milk, cheese or soy drink intake in serum and urine of healthy subjects. Interquartile range plotted with minimum, maximum, median (—) and outliers (◦), $n = 10$ participants. Left panel shows the intensity measured by LC-MS in serum or urine, 6 h postprandially and fasting 24 h after the ingestion of milk (orange), cheese (blue) or a soy drink (green). *, significant food effect using the Kruskall-Wallis test ($P < 0.05$, adjusted for multiple testing). Right panel shows the 6 h iAUC in serum or urine after milk, cheese or soy drink intake. *P* values adjusted for multiple testing using the Kruskall-Wallis test are indicated. If the Kruskal-Wallis test was significant ($P < 0.05$), pairwise comparisons were conducted using a Conover-Inman test. Different letters (a, b, c) denote significant differences based on a *P* value < 0.05. Detailed *P* values for the Kruskal-Wallis and Conover-Inman tests and for each time points are given in Supplemental Tables 2 and 3.

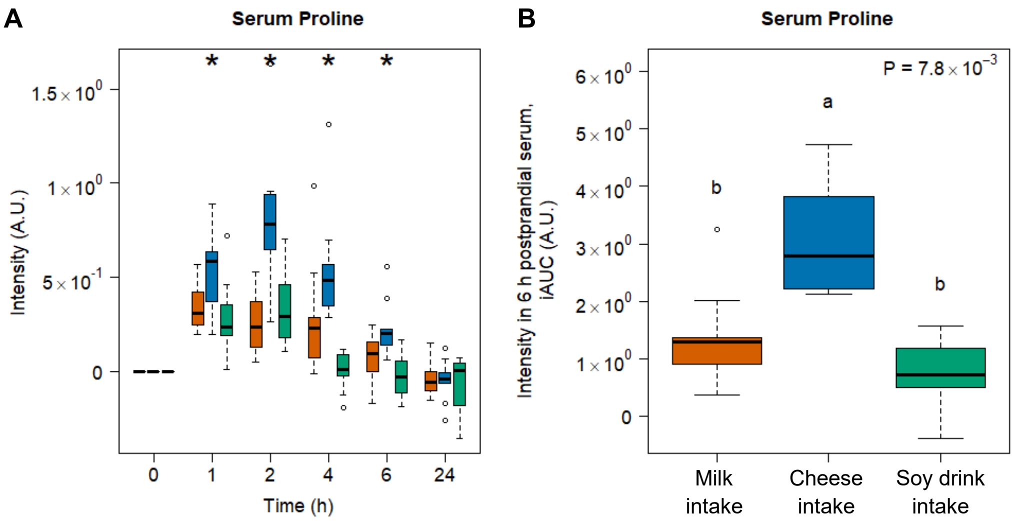

SUPPLEMENTAL FIGURE 15 Proline after milk, cheese or soy drink intake in serum of healthy subjects. Interquartile range plotted with minimum, maximum, median (—) and outliers (◦), n = 10 participants. (A) Intensity measured by LC-MS in serum, 6 h postprandially and fasting 24 h after the ingestion of milk (orange), cheese (blue) or a soy drink (green). *, significant food effect using the Kruskall-Wallis test (P < 0.05, adjusted for multiple testing). (B) 6 h iAUC in serum after milk, cheese or soy drink intake. P values adjusted for multiple testing using the Kruskall-Wallis test are indicated. If the Kruskal-Wallis test was significant (P < 0.05), pairwise comparisons were conducted using a Conover-Inman test. Different letters (a, b, c) denote significant differences based on a P value < 0.05. Detailed P values for the Kruskal-Wallis and Conover-Inman tests and for each time points are given in Supplemental Table 2.

SUPPLEMENTAL REFERENCES

1. Burton KJ, Krüger R, Scherz V, Münger LH, Picone G, Vionnet N, Bertelli C, Greub G, Capozzi F, Vergères G. Trimethylamine-N-Oxide Postprandial Response in Plasma and Urine Is Lower After Fermented Compared to Non-Fermented Dairy Consumption in Healthy Adults. Nutrients 2020; 12:234.
