# Supplementary material for: Identification of Milk and Cheese Intake Biomarkers in Healthy Adults Reveals High Interindividual Variability of Lewis System–Related Oligosaccharides
Source: J Nutr. 2020 Mar 4;150(5):1058–67. doi: 10.1093/jn/nxaa029 (PMC7198293; doi:10.1093/jn/nxaa029)
Supplement: nxaa029_Supplemental_Files [file nxaa029_supplemental_files.zip › G_Pimentel_JNutr_Supplemental_Methods_Revised_21_01_20.pdf]

Identification of milk and cheese intake biomarkers in healthy adults reveals high inter-individual variability of Lewis system related oligosaccharides.

Grégory Pimentel

Online Supplementary Material

SUPPLEMENTAL METHODS

Samples preparation for LC-MS metabolomics.

Blood samples were collected from participants in 4.7 ml vacutainer tubes (S-Monovette®-Sarstedt, Nümbrecht, Germany), incubated for 30 min at room temperature to allow clotting, and centrifuged at 1800 g for 10 min at 4 °C. The supernatants were aliquoted into smaller volumes and stored at -20 °C overnight before transfer to long-term storage at -80 °C. For LC-MS analysis, serum samples were allowed to thaw on ice, and were kept on ice along the preparation. In order to limit ion-suppression, phospholipids were removed from the samples with the use of a phospholipids removal device, which consists in a 96-well plate with a filter membrane (Phree®, Phenomenex Inc., Torrance, California, USA). Protein precipitation was obtained in the well plate with the addition 1:3 (vol/vol) of pre-chilled acetonitrile containing 1% (vol/vol) formic acid. The Phree® plate was vortexed for 2 min at 600 rpm and then centrifuged for 5 min at 500 g and at 4 °C. Samples were kept at 4 °C during the LC-MS analysis.

Urine samples were stored at 4 °C after collection, centrifuged (1800 g, 10 min, 4 °C), aliquoted, then stored overnight at -20 °C and transferred to -80 °C. For LC-MS analysis, urine samples were defrozen on ice, and diluted with Milli-Q water to a common refractive index of 1.3341 at 20°C, corresponding to a specific gravity of 1.00083 (refractometer RE40, Mettler Toledo, Switzerland). Quality control samples (QC), consisting in a pool of all serum or urine samples, were injected every five samples for signal drift correction. Blanks (ultrafiltered water) were as well regularly injected to account for contaminants.

LC-MS metabolomics analysis.

The chromatographic separation was performed using a high-pressure liquid chromatography system (HPLC, UltiMate™ 3000, Thermo Fisher Scientific™/Dionex™, Waltham, MA, USA) on a C18 hybrid silica column (Acquity UPLC HSS T3 1.8 µm 2.1 x 150 mm, Waters, UK), reverse phase. The mobile phase consisted in water containing 0.1% formic acid (A) and acetonitrile containing 0.1% formic acid (B). The elution gradient that was used (A:B, vol/vol)

was as follows: 95:5 at 0 min to 5:95 at 10 min; 5:95 from 10 to 20 min; 95:5 from 20 to 30 min. The flow rate was 0.4 ml/min. The HPLC system was coupled to a quadrupole time-of-flight mass spectrometer (QTOF-MS, maXis™ 4G+, Bruker Daltonik GmbH, Bremen, Germany). The MS electrospray interface operated in positive ion mode. Electrospray voltage was set to 4.5 kV, end plate offset to 500 V, capillary voltage to 3400 V, nitrogen flow set to 4ml/min at 200°C. The spectra acquisition rate was set to 1 Hz in profile mode, spectra were recorded from m/z 75 to m/z 1500 at a resolution of 80,000 FWHM. An internal calibration was used with 50:50 water/isopropanol containing 0.2% (vol/vol) formic acid and 1% (vol/vol) 1M sodium hydroxide. All solvents and reagents were LC-MS grade (Sigma-Aldrich GmbH, Stettlen, Switzerland). As the analysis is semi-quantitative, the metabolites concentrations mentioned in the text refer to relative concentrations, determined from the metabolites' peak area (arbitrary unit).

Signal drift correction and data filtering

The QC-based robust locally estimated scatterplot smoothing signal correction method was applied for signal drift correction (1) using R (v.3.1.2; R Foundation for Statistical Computing, Vienna, Austria).

Metabolites with poor repeatability, *i.e.* detected in < 50 % of QCs, were removed, as well as metabolites with a relative standard deviation > 30 % in the QC samples. Features that had a median in the QC samples that was < 3 times higher than the median calculated for the blanks were also excluded.

Standards suppliers for identified BFIs

Blood group H disaccharide	Carbosynth, Compton, Newbury, UK
Lewis A trisaccharide	Carbosynth, Compton, Newbury, UK
Galactonic acid / Gluconic acid	MSML kit (IROA Technologies, LLC, Bolton, MA; Gainesville, FL)
Aminoadipic acid	MSML kit (IROA Technologies, LLC, Bolton, MA; Gainesville, FL)
Citrulline	MSML kit (IROA Technologies, LLC, Bolton, MA; Gainesville, FL)
Valyl-Threonine	Synpeptide Co Ltd, Shanghai, China
Phenylalanyl-Proline	Synpeptide Co Ltd, Shanghai, China
Indolelactic acid	Sigma-Aldrich Chemie GmbH (Buchs, Switzerland)
Proline	MSML kit (IROA Technologies, LLC, Bolton, MA; Gainesville, FL)

SUPPLEMENTAL REFERENCES

1. Dunn WB, Broadhurst D, Begley P, Zelena E, Francis-McIntyre S, Anderson N, Brown M, Knowles JD, Halsall A, Haselden JN, et al. Procedures for large-scale metabolic profiling of serum and plasma using gas chromatography and liquid chromatography coupled to mass spectrometry. Nat Protoc. 2011;6:1060-83.
