# Supplementary material for: Identification of Milk and Cheese Intake Biomarkers in Healthy Adults Reveals High Interindividual Variability of Lewis System–Related Oligosaccharides
Source: J Nutr. 2020 Mar 4;150(5):1058–67. doi: 10.1093/jn/nxaa029 (PMC7198293; doi:10.1093/jn/nxaa029)
Supplement: nxaa029_Supplemental_Files [file nxaa029_supplemental_files.zip › G_Pimentel_JNutr_Supplemental_Table 1_Revised_21_01_20.pdf]

SUPPLEMENTAL TABLE 1 Details for metabolites discriminant for milk, cheese or soy drink intake in postprandial serum and/or 24 h fasting serum of healthy adults.[1]

								Postprandial serum		Fasting serum	
**m/z**	**Charge**	**Adducts**	**Neutral mass (Da)**	**Retention time (min)**	**HMDB / NIST ref.**	**Id. level [2]**	**Name / Class**	**Food source**	**VIP score**	**Food source**	**VIP score**
231.1337659	1	M+H, 2M+Na, 2M+H	230.1264895	1.06	HMDB28876	2	Hydroxyprolyl-Valine/Valyl-Hydroxyproline	Cheese	4.7	-	-
368.1783756	1	-	-	1.06	-	3	Direct parent: Phenothiazines	Cheese	4.6	-	-
211.0337069	1	-	-	6.25	-	4	Unknown	Cheese	4.1	-	-
242.1244464	1	-	-	1.04	-	3	Super class: Organoheterocyclic compounds	Cheese	4.0	-	-
253.1154058	1	M+H, M+2Na-H	252.1081294	1.07	-	4	Unknown	Cheese	4.0	-	-
**219.1337848**	**1**	**M+H, 2M+H**	**218.1265084**	**1.18**	**HMDB29137**	**1**	**Valyl-Threonine**	**Cheese**	**3.9**	**-**	**-**
484.0949791	1	-	-	1.07	-	4	Unknown	Cheese	3.8	-	-
189.0513679	1	M+ACN+H	147.0175446	6.25	HMDB34370	2	Ibervirin	Cheese	3.5	-	-
331.1315569	1	M+NH4	313.0977342	1.28	HMDB14683	2	Amoxapine	Cheese	3.5	-	-
373.0737487	1	M+H, M+Na, M+2Na-H	350.0845281	1.06	-	4	Unknown	Cheese	3.5	-	-
311.0736241	1	M+H, M+Na, M+2Na-H	310.0658031	1.06	-	4	Unknown	Cheese	3.1	-	-
247.139596	1	M+NH4	229.1057733	1.06	HMDB28739	2	Asparagyl-Proline / Prolyl-Asparagine	Cheese	3.0	-	-
369.0317816	1	M+H, M+Na	368.0245052	1.07	HMDB00218	2	Orotidylic acid	Cheese	2.9	-	-
143.0812742	1	M+ACN+H, 2M+ACN+H	101.0474487	1.09	-	4	Unknown	Cheese	2.8	Soy drink	3.0
257.1489722	1	M+H, M+Na	256.1416957	6.02	-	4	Unknown	Cheese	2.7	-	-
**116.0706403**	**1**	**M+H, M+Na, M+2Na-H**	**115.0633639**	**1.07**	**HMDB00162**	**1**	**Proline**	**Cheese**	**2.7**	**-**	**-**
378.1483466	1	-	-	1.00	-	4	Unknown	Cheese	2.6	-	-
256.0063983	1	-	-	1.02	-	4	Unknown	Cheese	2.6	-	-
486.9454704	1	-	-	1.06	-	4	Unknown	Cheese	2.6	-	-
294.4858405	1	M+H-H2O, M+H	311.4891287	0.86	-	4	Unknown	Cheese	2.5	-	-
426.9900037	1	-	-	1.06	-	4	Unknown	Cheese	2.5	-	-
746.8959752	1	-	-	0.90	-	4	Unknown	Cheese	2.5	-	-
302.120613	1	M+Na, M+2Na-H, M+H	279.1313924	1.07	HMDB37844	2	N-(1-Deoxy-1-fructosyl)valine	Cheese	2.4	-	-
189.1229835	1	-	-	1.11	-	4	Unknown	Cheese	2.4	-	-

[1] Metabolites were considered as discriminant when VIP scores > 1.5 based on one-by-two OLPS-DA, *n* = 10 participants. Identified metabolites considered as putative BFIs are in bold. OPLS-DA, Orthogonal partial least squares discriminant analysis; VIP, variable importance in projection for the predictive component.
[2] Identification levels: level 1, compounds were identified by comparison to a pure reference (based on spectral data and retention time (RT), with 10% as a retention time difference threshold); level 2, without chemical standards, based upon spectral data (including fragmentation pattern when available); level 3: putatively characterized compound classes; level 4: unknown compound. For level 3 identification, chemical taxonomie as reported by the human metabolome database is indicated.

m/z	Charge	Adducts	Neutral mass (Da)	Retention time (min)	HMDB / NIST ref.	Id. level	Name / Class	Postprandial serum Food source	Postprandial serum VIP score	Fasting serum Food source	Fasting serum VIP score
147.0439855	1	-	-	1.28	-	4	Unknown	Cheese	2.4	-	-
123.0439541	1	M+H-H2O, M+NH4	140.0472423	1.28	-	4	Unknown	Cheese	2.4	-	-
274.0914043	1	M+H-H2O, M+NH4, M+Na, M+ACN+Na	210.073342	1.16	-	3	Sub class: Carbohydrates and carbohydrate conjugates	Cheese	2.3	-	-
182.0811114	1	M+H, M+Na, 2M+H	181.0738349	1.28	-	4	Unknown	Cheese	2.3	-	-
165.0544813	1	-	-	1.28	-	4	Unknown	Cheese	2.3	-	-
384.0898828	1	M+H-H2O, M+H	383.0796835	1.26	-	4	Unknown	Cheese	2.3	-	-
428.9876921	1	-	-	1.06	-	4	Unknown	Cheese	2.3	-	-
658.3641428	2	-	-	6.70	-	4	Unknown	Cheese	2.2	Soy drink	1.7
279.1003687	1	-	-	1.28	-	4	Unknown	Cheese	2.2	-	-
498.3411364	1	M+NH4	480.3073137	10.73	HMDB30180	2	Crustecdysone	Cheese	2.2	-	-
136.0756535	1	-	-	1.28	-	4	Unknown	Cheese	2.2	-	-
150.0582856	1	M+H, M+NH4, M+H-H2O	132.0244185	1.28	HMDB59667	2	3-Methyl sulfolene	Cheese	2.2	-	-
329.0333047	1	-	-	1.32	-	4	Unknown	Cheese	2.2	-	-
279.9919366	1	M+H, M+2Na-H	235.0220988	1.06	-	4	Unknown	Cheese	2.2	-	-
706.8848238	1	-	-	0.85	-	4	Unknown	Cheese	2.2	-	-
313.07099	1	-	-	1.07	-	4	Unknown	Cheese	2.2	-	-
268.9593944	1	-	-	0.88	-	4	Unknown	Cheese	2.1	-	-
305.1449138	1	M+NH4, M+2Na-H	260.1768643	1.21	-	3	Super class: Lipids and lipid-like molecules	Cheese	2.1	-	-
245.112726	1	-	-	1.23	-	4	Unknown	Cheese	2.1	-	-
229.5069772	2	-	-	0.86	-	4	Unknown	Cheese	2.0	-	-
342.9883026	1	-	-	0.95	-	4	Unknown	Cheese	2.0	-	-
508.7878105	1	-	-	0.93	-	4	Unknown	Cheese	2.0	-	-
235.1648797	1	M+H-H2O, M+H, M+Na, M+2Na-H	234.1576033	1.26	-	4	Unknown	Cheese	2.0	-	-
458.0876182	1	M+NH4, M+2Na-H	440.0537927	1.00	-	4	Unknown	Cheese	2.0	-	-
243.1332959	1	-	-	4.98	-	4	Unknown	Cheese	2.0	-	-
594.8637662	2	-	-	0.90	-	3	Sub class: Glycosphingolipids	Cheese	2.0	-	-
329.0720281	1	-	-	1.28	-	3	Direct parent: Thiosulfinic acid esters	Cheese	2.0	-	-
226.0446121	1	M+H-H2O, M+Na	243.0479003	2.22	-	4	Unknown	Cheese	2.0	-	-
647.1786185	1	-	-	1.28	-	3	Super class: Phenylpropanoids and polyketides	Cheese	2.0	-	-
576.7735936	1	-	-	0.93	-	4	Unknown	Cheese	2.0	-	-
255.9661924	1	M+H, M+Na	254.9589159	1.06	-	4	Unknown	Cheese	1.9	-	-
479.1506163	1	-	-	1.28	-	4	Unknown	Cheese	1.9	-	-
118.0862793	1	M+H, M+Na	117.0790028	1.26	-	4	Unknown	Cheese	1.9	-	-
**263.1386057**	**1**	**M+H**	**262.1313298**	**5.08**	**HMDB11177**	**1**	**L-phenylalanyl-L-proline**	**Cheese**	**1.9**	**-**	**-**
371.0292339	1	M+H, M+Na, M+2Na-H	348.0400133	1.07	-	4	Unknown	Cheese	1.9	-	-

m/z	Charge	Adducts	Neutral mass (Da)	Retention time (min)	HMDB / NIST ref.	Id. level	Name / Class	Postprandial serum		Fasting serum	
								Food source	VIP score	Food source	VIP score
119.0490528	1	M+H-H2O, M+NH4, M+ACN+H, 2M+H, 2M+ACN+Na	136.052341	1.28	-	4	Unknown	Cheese	1.9	-	-
186.1120943	1	M+H-H2O, M+ACN+Na	203.1153825	1.28	HMDB00201	2	Acetyl-L-carnitine	Cheese	1.9	-	-
570.9884396	1	M+H-H2O, M+2Na-H	587.9917279	0.88	-	4	Unknown	Cheese	1.9	-	-
**206.080798**	**1**	**M+NH4, M+2Na-H, M+H, M+Na**	**205.0738319**	**6.55**	**HMDB00671**	**1**	**Indolelactic acid**	**Cheese**	**1.8**	**-**	**-**
**162.0756725**	**1**	**M+H, M+2Na-H, M+Na, M+H-H2O**	**161.0683961**	**1.07**	**HMDB00510**	**1**	**Aminoadipic acid**	**Cheese**	**1.8**	**-**	**-**
332.0840125	1	-	-	1.02	-	4	Unknown	Cheese	1.8	-	-
253.0466617	1	M+H-H2O, M+H	270.0499499	0.88	-	4	Unknown	Cheese	1.8	-	-
400.1290973	1	M+H, M+NH4	399.1218209	1.00	-	4	Unknown	Cheese	1.8	-	-
595.796183	1	-	-	0.93	-	4	Unknown	Cheese	1.8	-	-
132.1019883	1	M+H-H2O, M+ACN+Na	149.1052765	1.30	HMDB32538	2	Triethanolamine	Cheese	1.8	-	-
383.0043466	1	M+H-H2O, M+2Na-H	400.0076348	0.88	-	4	Unknown	Cheese	1.8	-	-
493.2779253	2	M+2H, M+NH4, M+2Na	984.5378007	6.69	-	4	Unknown	Cheese	1.8	-	-
342.2632058	1	-	-	9.98	-	3	Super class: Lipids and lipid-like molecules	Cheese	1.8	-	-
373.0437587	1	M+NH4, M+ACN+Na	355.0099332	0.88	-	4	Unknown	Cheese	1.8	-	-
**176.1026481**	**1**	**M+H, M+2Na-H, M+Na**	**175.0953716**	**0.99**	**HMDB00904**	**1**	**Citrulline**	**Cheese**	**1.8**	**-**	**-**
275.0907714	1	M+H-H2O, M+ACN+H	233.0571533	2.38	-	4	Unknown	Cheese	1.8	-	-
303.4911346	2	-	-	0.86	-	4	Unknown	Cheese	1.8	-	-
246.169557	1	-	-	4.80	-	4	Unknown	Cheese	1.8	-	-
194.0062856	1	-	-	1.02	-	4	Unknown	Cheese	1.8	-	-
331.0292812	1	M+Na	308.0400629	0.79	HMDB01409	2	2'-Deoxyuridine 5'-monophosphate	Cheese	1.8	-	-
373.8805881	1	M+H, 2M+ACN+H	165.9261658	1.06	-	4	Unknown	Cheese	1.8	-	-
677.8278774	1	-	-	0.93	-	4	Unknown	Cheese	1.8	-	-
649.7395595	1	-	-	0.93	-	4	Unknown	Cheese	1.7	-	-
384.1367735	1	M+H-H2O, M+2Na-H	339.1637888	1.26	-	4	Unknown	Cheese	1.7	-	-
457.0083925	1	-	-	0.88	-	4	Unknown	Cheese	1.7	-	-
592.9829543	1	M+H-H2O, M+2Na-H	609.9862426	0.90	-	4	Unknown	Cheese	1.7	-	-
448.0854637	1	M+NH4, M+2Na-H	403.1119601	1.30	HMDB37550	2	2,4-Dihydroxy-7,8-dimethoxy-2H-1,4-benzoxazin-3(4H)-one 2-glucoside	Cheese	1.7	-	-
225.0842977	1	-	-	1.06	-	3	Sub class: Amino acids, peptides, and analogues	Cheese	1.7	-	-
516.0456587	1	-	-	1.00	-	4	Unknown	Cheese	1.7	-	-
130.0861536	1	-	-	0.86	-	4	Unknown	Cheese	1.7	-	-
596.917456	1	-	-	0.92	-	4	Unknown	Cheese	1.7	-	-
541.8533504	1	-	-	0.93	-	4	Unknown	Cheese	1.7	-	-
795.8167771	2	-	-	0.88	-	4	Unknown	Cheese	1.7	-	-

m/z	Charge	Adducts	Neutral mass (Da)	Retention time (min)	HMDB / NIST ref.	Id. level	Name / Class	Postprandial serum		Fasting serum	
								Food source	VIP score	Food source	VIP score
313.9244809	1	M+H, M+Na	312.9172044	1.06	-	4	Unknown	Cheese	1.7	-	-
340.0971617	1	M+ACN+H, M+2Na-H	295.1261272	1.06	-	4	Unknown	Cheese	1.7	-	-
466.3153824	1	M+H-H2O, M+H, M+NH4, M+Na, M+2Na-H, 2M+H, 2M+NH4, 2M+Na	465.3081059	9.35	-	3	Direct parent: Glycinated bile acids and derivatives	Cheese	1.7	-	-
253.9689271	1	M+H, M+Na	252.9616507	1.06	-	4	Unknown	Cheese	1.6	-	-
644.9147715	1	M+H-H2O, M+H	643.8996178	0.90	-	4	Unknown	Cheese	1.6	-	-
382.4176992	2	-	-	0.81	-	4	Unknown	Cheese	1.6	-	-
223.9846331	1	-	-	0.99	-	4	Unknown	Cheese	1.6	-	-
368.9565377	1	-	-	1.06	-	4	Unknown	Cheese	1.6	-	-
740.4530993	1	-	-	6.93	-	4	Unknown	Cheese	1.6	-	-
136.039133	1	-	-	4.09	-	4	Unknown	Cheese	1.6	-	-
246.0263987	1	-	-	5.24	-	4	Unknown	Cheese	1.6	-	-
170.0144394	1	-	-	1.33	-	4	Unknown	Cheese	1.6	-	-
560.8452016	1	-	-	0.97	-	4	Unknown	Cheese	1.6	-	-
796.9446491	1	-	-	0.90	-	4	Unknown	Cheese	1.6	-	-
135.1093334	1	M+ACN+Na, 2M+ACN+Na	71.09356362	1.30	-	4	Unknown	Cheese	1.6	-	-
626.3518607	1	M+H, M+Na	625.3445843	9.27	HMDB02579	2	Glycochenodeoxycholic acid 3-glucuronide	Cheese	1.6	-	-
350.1088895	1	M+H, M+Na, 2M+H	349.1016131	1.28	-	4	Unknown	Cheese	1.6	-	-
376.0709492	1	-	-	1.06	-	4	Unknown	Cheese	1.6	-	-
220.0756301	1	M+ACN+H	178.0418068	4.14	HMDB00078	2	Cys-Gly/Gly-Cys	Cheese	1.6	-	-
678.9082419	1	-	-	0.90	-	4	Unknown	Cheese	1.6	-	-
305.0968454	1	-	-	1.30	-	4	Unknown	Cheese	1.6	-	-
321.0339359	1	-	-	0.88	-	4	Unknown	Cheese	1.6	-	-
339.2667791	1	-	-	10.75	-	3	Direct parent: Very long-chain fatty acids	Cheese	1.6	-	-
264.2675554	1	M+H, M+NH4	246.2342759	11.67	-	4	Unknown	Cheese	1.5	-	-
283.1196344	1	-	-	15.07	-	4	Unknown	Cheese	1.5	Soy drink	1.8
430.2940842	1	M+H-H2O, M+H	429.28761	9.35	-	4	Unknown	Cheese	1.5	-	-
518.9787565	1	M+ACN+H, M+2Na-H, 2M+H	258.9873931	0.88	-	4	Unknown	Cheese	1.5	-	-
311.1231694	1	-	-	1.28	-	4	Unknown	Cheese	1.5	-	-
298.1312495	1	-	-	1.26	-	4	Unknown	Cheese	1.5	-	-
303.0860061	1	-	-	7.09	-	4	Unknown	Cheese	1.5	Soy drink	2.0
563.3209613	2	-	-	6.34	-	4	Unknown	Cheese	1.5	-	-
643.3782579	1	-	-	9.13	-	3	Super class: Lipids and lipid-like molecules	Cheese	1.5	-	-
344.2534479	1	-	-	6.34	-	4	Unknown	Milk	9.7	-	-
**530.2068678**	**1**	**M+H, M+Na**	**529.1995914**	**1.14**	**HMDB02060**	**1**	**Lewis a trisaccharide**	**Milk**	**9.1**	**-**	**-**
186.1124015	1	-	-	6.09	-	4	Unknown	Milk	7.4	-	-

								Postprandial serum		Fasting serum	
**m/z**	**Charge**	**Adducts**	**Neutral mass (Da)**	**Retention time (min)**	**HMDB / NIST ref.**	**Id. level #**	**Name / Class**	**Food source**	**VIP score**	**Food source**	**VIP score**
**349.1099857**	**1**	**M+Na**	**326.1207674**	**1.07**	**HMDB06590**	**1**	**Blood group H disaccharide**	**Milk**	**4.3**	**-**	**-**
205.1179388	1	M+H	182.1287205	1.07	HMDB29136	1	Valyl-Serine	Milk	4.0	-	-
203.0142266	1	-	-	1.06	-	4	Unknoswn	Milk	3.4	-	-
219.0467182	1	M+Na	196.0574999	1.02	HMDB00565	3	Galactonic acid/Gluconic acid	Milk	3.3	-	-
467.2564904	2	-	-	5.00	-	4	Unknown	Milk	3.1	-	-
289.2155292	1	M+H-H2O, M+H	288.2081044	10.70	-	4	Unknown	Milk	2.7	-	-
615.3721464	1	M+H-H2O, M+H, M+NH4	614.3648699	8.99	-	4	Unknown	Milk	2.5	-	-
302.2318741	1	-	-	8.08	-	4	Unknown	Milk	2.5	-	-
671.3819496	2	-	-	7.26	-	4	Unknown	Milk	2.4	-	-
583.3298441	2	-	-	7.02	-	4	Unknown	Milk	2.4	-	-
779.8069018	1	-	-	0.92	-	4	Unknown	Milk	2.3	Soy drink	2.5
539.2190482	1	-	-	5.75	-	4	Unknown	Milk	2.3	-	-
265.2012485	1	-	-	1.30	-	3	Direct parent: Alkylthiols	Milk	2.3	-	-
133.0970057	1	M+H-H2O, M+H	132.0897747	0.86	HMDB00214	2	Ornithine	Milk	2.2	-	-
437.1641845	1	-	-	9.49	-	3	Super class: Lipids and lipid-like molecules	Milk	2.2	-	-
428.997433	1	-	-	0.86	-	4	Unknown	Milk	2.1	-	-
510.9801444	1	-	-	0.88	-	4	Unknown	Milk	2.1	-	-
528.3184868	2	M+H, M+H+NH4	1037.595874	8.66	-	4	Unknown	Milk	2.1	-	-
556.9728378	1	-	-	0.88	-	4	Unknown	Milk	2.0	-	-
689.4051672	1	-	-	6.04	-	4	Unknown	Milk	2.0	-	-
606.3292333	2	-	-	5.73	-	4	Unknown	Milk	2.0	-	-
335.2205774	1	-	-	9.28	-	4	Unknown	Milk	1.9	-	-
375.0055406	1	M+NH4, 2M+H	186.997715	0.90	-	4	Unknown	Milk	1.9	-	-
630.4198239	1	M+H, M+NH4	612.3855569	10.19	HMDB10355	2	Cholestane-3,7,12,25-tetrol-3-glucuronide	Milk	1.9	-	-
290.1339083	1	M+H-H2O, M+2Na-H	307.1371966	1.30	-	4	Unknown	Milk	1.8	-	-
373.0254531	1	-	-	1.09	-	4	Unknown	Milk	1.8	-	-
562.7630934	1	-	-	0.95	-	4	Unknown	Milk	1.8	-	-
271.9641759	1	-	-	0.99	-	4	Unknown	Milk	1.8	-	-
709.0732635	2	-	-	1.00	-	4	Unknown	Milk	1.8	-	-
313.0352781	1	M+H, 2M+ACN+H	312.0280017	0.90	-	4	Unknown	Milk	1.8	-	-
352.0463658	1	-	-	6.37	-	4	Unknown	Milk	1.8	-	-
307.0184106	1	M+H-H2O, M+Na	284.025455	0.88	-	4	Unknown	Milk	1.8	-	-
347.1983773	2	M+2H, M+H+NH4	692.3822017	5.50	-	4	Unknown	Milk	1.8	-	-
263.1961272	1	M+H, M+ACN+H	221.162095	1.30	-	4	Unknown	Milk	1.8	-	-
578.9674656	1	-	-	0.90	-	4	Unknown	Milk	1.7	-	-
660.372658	1	M+H, M+NH4, M+Na	659.3653815	11.58	-	4	Unknown	Milk	1.7	-	-
464.1903993	1	M+H, M+Na	463.1831229	7.52	-	4	Unknown	Milk	1.7	-	-
235.0594273	1	-	-	0.86	-	4	Unknown	Milk	1.7	-	-
581.3261128	2	M+2H, M+2Na	1160.637673	7.00	-	4	Unknown	Milk	1.7	-	-

								Postprandial serum		Fasting serum	
**m/z**	**Charge**	**Adducts**	**Neutral mass (Da)**	**Retention time (min)**	**HMDB / NIST ref.**	**Id. level** #	**Name / Class**	**Food source**	**VIP score**	**Food source**	**VIP score**
432.7412408	2	-	-	5.87	-	3	Direct parent: Phosphatidylglycerophosphates	Milk	1.6	-	-
647.3651337	2	M+2H, M+2Na	1292.715715	7.17	-	4	Unknown	Milk	1.6	-	-
686.4050622	2	M+2H, M+H+NH4, M+H+Na	1353.769024	7.24	-	4	Unknown	Milk	1.6	-	-
598.353058	2	-	-	7.00	-	4	Unknown	Milk	1.6	-	-
774.4572588	2	M+2H	1546.899965	7.44	HMDB11878	2	Ganglioside GM1 (d18:0/18:0)	Milk	1.6	-	-
187.126197	1	-	-	9.49	-	3	Class: Azoles	Milk	1.6	-	-
654.8613876	2	-	-	0.90	-	4	Unknown	Milk	1.6	-	-
720.8922498	1	-	-	0.90	-	4	Unknown	Milk	1.6	-	-
245.048019	1	M+H	244.0407431	0.90	HMDB59983	2	4-phenylbutanic acid-O-sulphate	Milk	1.6	-	-
475.1699813	1	M+ACN+Na, 2M+H	237.0812313	8.10	-	4	Unknown	Milk	1.6	-	-
519.1274409	1	-	-	12.32	-	4	Unknown	Milk	1.6	-	-
728.4089818	2	M+2H, M+2Na	1454.803411	6.98	-	4	Unknown	Milk	1.6	-	-
492.1855378	1	M+NH4, M+Na	474.1517122	7.21	-	4	Unknown	Milk	1.6	-	-
538.3572958	1	-	-	10.92	-	4	Unknown	Milk	1.6	Soy drink	1.5
539.3039637	2	-	-	6.86	-	4	Unknown	Milk	1.6	-	-
189.1229962	1	-	-	1.26	-	4	Unknown	Milk	1.5	-	-
347.9851299	1	M+NH4	329.9513072	1.06	40180049	2	Tienilic acid	Milk	1.5	-	-
118.0609564	1	-	-	1.00	-	3	Class: Carboxylic acids and derivatives	Milk	1.5	-	-
445.1693862	1	M+H, M+NH4, M+Na	444.1621097	9.28	-	4	Unknown	Milk	1.5	-	-
546.7789954	2	-	-	5.45	-	4	Unknown	Milk	1.5	-	-
430.263891	2	-	-	5.87	-	4	Unknown	Milk	1.5	-	-
627.3558625	2	-	-	7.14	-	4	Unknown	Milk	1.5	-	-
310.2005408	1	-	-	7.42	-	4	Unknown	Milk	1.5	-	-
447.0912218	1	M+H, M+NH4, M+Na	446.0839454	6.69	-	4	Unknown	Soy drink	9.9	-	-
431.0963475	1	-	-	5.50	-	4	Unknown	Soy drink	9.5	-	-
527.0478547	1	M+H-H2O	544.0516927	5.24	HMDB37596	2	8-Hydroxyluteolin 8-glucoside 3'-sulfate	Soy drink	9.1	Soy drink	4.1
447.0912465	1	-	-	6.32	-	4	Unknown	Soy drink	8.9	-	-
335.0213775	1	-	-	6.69	-	4	Unknown	Soy drink	8.8	-	-
431.0962596	1	-	-	5.97	-	4	Unknown	Soy drink	8.6	-	-
194.0655184	1	-	-	11.81	-	4	Unknown	Soy drink	7.3	-	-
346.2216359	1	M+ACN+H	304.1878126	6.23	107645	2	4-(2,3-Dihydroxypropyl) 2-(2-methyl-4,4-dimethylpentyl)succinate	Soy drink	3.5	-	-
344.2787943	1	-	-	10.42	-	3	Super class: Lipids and lipid-like molecules	Soy drink	3.3	-	-
882.3557819	1	-	-	7.70	-	4	Unknown	Soy drink	3.2	-	-
283.1280565	1	-	-	6.04	-	4	Unknown	Soy drink	3.2	-	-
295.2990102	1	-	-	12.77	-	3	Super class: Lipids and lipid-like molecules	Soy drink	3.2	-	-
794.5721088	1	-	-	7.70	-	3	Direct parent: Phosphatidylcholines	Soy drink	3.2	-	-

								Postprandial serum		Fasting serum	
**m/z**	**Charge**	**Adducts**	**Neutral mass (Da)**	**Retention time (min)**	**HMDB / NIST ref.**	**Id. level #**	**Name / Class**	**Food source**	**VIP score**	**Food source**	**VIP score**
756.4015294	1	M+NH4, M+ACN+Na	738.3677039	7.70	-	4	Unknown	Soy drink	3.2	-	-
370.1880371	1	M+H-H2O, M+H	369.1810487	16.52	HMDB15288	2	Trimetrexate	Soy drink	3.2	-	-
882.6343426	1	-	-	7.70	-	4	Unknown	Soy drink	3.1	-	-
836.2329963	1	-	-	7.70	-	4	Unknown	Soy drink	3.1	-	-
794.5219686	1	-	-	7.70	-	3	Sub class: Amino acids, peptides, and analogues	Soy drink	3.1	-	-
835.9692441	1	-	-	7.70	-	4	Unknown	Soy drink	3.1	-	-
662.3113967	1	M+H-H2O, M+2Na-H	679.3146849	7.70	-	4	Unknown	Soy drink	3.0	-	-
836.2858323	1	-	-	7.70	-	3	Super class: Phenylpropanoids and polyketides	Soy drink	3.0	-	-
835.9164217	1	-	-	7.70	-	4	Unknown	Soy drink	3.0	-	-
318.1904828	1	-	-	4.84	-	4	Unknown	Soy drink	2.9	-	-
265.9296221	1	M+H-H2O, M+H	282.9329104	0.83	-	4	Unknown	Soy drink	2.8	-	-
756.4492221	1	M+NH4, M+ACN+Na	738.4153966	7.70	-	3	Sub class: Steroidal glycosides	Soy drink	2.8	-	-
756.5448034	1	M+NH4, M+2Na-H	711.5787514	7.70	-	4	Unknown	Soy drink	2.8	-	-
691.0637307	1	-	-	7.71	-	4	Unknown	Soy drink	2.7	-	-
756.5925076	1	M+NH4, M+2Na-H	711.6245589	7.70	-	4	Unknown	Soy drink	2.7	-	-
756.640244	1	M+NH4, M+2Na-H	711.6691201	7.70	-	4	Unknown	Soy drink	2.7	-	-
690.6715737	1	-	-	7.71	-	4	Unknown	Soy drink	2.6	-	-
488.9898829	1	-	-	1.06	-	4	Unknown	Soy drink	2.6	-	-
756.4970403	1	M+NH4, M+2Na-H	711.5338062	7.71	-	4	Unknown	Soy drink	2.5	-	-
400.1861785	1	-	-	5.17	-	4	Unknown	Soy drink	2.5	-	-
175.1074366	1	M+H-H2O, M+H	174.1001769	1.13	-	3	Sub class: Amino acids, peptides, and analogues	Soy drink	2.5	-	-
502.2894699	1	-	-	13.75	-	4	Unknown	Soy drink	2.4	-	-
412.3046065	1	-	-	9.63	-	4	Unknown	Soy drink	2.3	-	-
593.3323756	1	-	-	15.12	-	4	Unknown	Soy drink	2.2	-	-
625.352224	2	M+2H, M+H+NH4, M+2Na	1248.689895	7.12	-	4	Unknown	Soy drink	2.2	-	-
458.2011983	1	-	-	7.02	-	4	Unknown	Soy drink	2.2	Soy drink	1.5
882.4672692	1	-	-	7.70	-	4	Unknown	Soy drink	2.1	-	-
340.140791	1	-	-	14.30	-	4	Unknown	Soy drink	2.0	-	-
389.2671167	1	-	-	11.31	-	4	Unknown	Soy drink	2.0	-	-
451.2569054	1	-	-	16.01	-	3	Sub class: Amino acids, peptides, and analogues	Soy drink	2.0	-	-
465.171897	1	-	-	1.28	-	4	Unknown	Soy drink	1.9	-	-
328.2475002	1	-	-	9.13	-	4	Unknown	Soy drink	1.9	-	-
418.3204969	1	2M+ACN+Na	177.1523659	10.96	4107986	2	N,N-Diisopropylaniline	Soy drink	1.9	-	-
1039.125954	2	-	-	1.00	-	4	Unknown	Soy drink	1.9	-	-
509.3099012	1	-	-	16.01	-	4	Unknown	Soy drink	1.9	-	-
359.0905852	1	-	-	0.97	-	4	Unknown	Soy drink	1.9	-	-

								Postprandial serum		Fasting serum	
**m/z**	**Charge**	**Adducts**	**Neutral mass (Da)**	**Retention time (min)**	**HMDB / NIST ref.**	**Id. level #**	**Name / Class**	**Food source**	**VIP score**	**Food source**	**VIP score**
530.898427	1	-	-	0.81	-	4	Unknown	Soy drink	1.9	-	-
620.798987	1	-	-	0.95	-	4	Unknown	Soy drink	1.8	Soy drink	2.0
221.0233178	1	-	-	2.31	-	4	Unknown	Soy drink	1.8	-	-
749.9033701	1	-	-	0.90	-	4	Unknown	Soy drink	1.7	-	-
486.1139306	1	-	-	1.28	-	4	Unknown	Soy drink	1.7	-	-
131.048978	1	-	-	6.90	-	4	Unknown	Soy drink	1.6	-	-
370.2115925	1	-	-	12.49	-	4	Unknown	Soy drink	1.6	-	-
335.1507511	1	-	-	16.01	-	4	Unknown	Soy drink	1.6	-	-
1157.137111	1	-	-	1.00	-	4	Unknown	Soy drink	1.6	-	-
528.3071229	1	M+H, M+Na	527.2998464	13.12	-	3	Sub class: Glycerophosphoethanolamines	Soy drink	1.6	-	-
307.1502438	1	-	-	1.28	-	4	Unknown	Soy drink	1.6	-	-
478.2920625	1	M+H-H2O, M+H, M+Na, M+2Na-H, 2M+H	477.284786	12.75	-	3	Sub class: Glycerophosphoethanolamines	Soy drink	1.6	-	-
264.9724219	1	-	-	0.97	-	4	Unknown	Soy drink	1.6	-	-
368.2786334	1	-	-	10.35	-	4	Unknown	Soy drink	1.6	-	-
600.8303167	2	M+2H, M+H+Na, M+2Na	1155.683215	7.00	-	4	Unknown	Soy drink	1.6	-	-
317.2680749	1	-	-	15.77	-	4	Unknown	Soy drink	1.6	-	-
500.92785	1	M+NH4, 2M+H	249.9591698	1.06	-	4	Unknown	Soy drink	1.6	-	-
680.9449091	1	-	-	1.02	-	4	Unknown	Soy drink	1.6	-	-
594.1776428	1	M+H-H2O, M+ACN+Na	530.1606953	1.30	-	4	Unknown	Soy drink	1.5	-	-
450.3206026	1	M+H-H2O, M+H, M+Na, M+2Na-H, 2M+H, 2M+NH4	449.3133262	11.04	-	3	Sub class: Bile acids, alcohols and derivatives	Soy drink	1.5	-	-
260.1851241	1	-	-	5.99	-	4	Unknown	Soy drink	1.5	-	-
452.2760592	1	-	-	12.21	-	4	Unknown	Soy drink	1.5	-	-
235.0564127	1	-	-	0.93	-	3	Sub class: Amino acids, peptides, and analogues	Soy drink	1.5	-	-
834.3654212	1	M+H, M+2H	833.3582062	6.58	-	4	Unknown	-	-	Soy drink	5.5
423.062926	1	-	-	1.07	-	4	Unknown	-	-	Soy drink	2.8
574.3432371	1	-	-	5.45	-	3	Super class: Lipids and lipid-like molecules	-	-	Soy drink	2.5
681.8311399	1	-	-	0.90	-	4	Unknown	-	-	Soy drink	2.4
318.2528749	1	-	-	9.18	-	4	Unknown	-	-	Soy drink	2.4
881.7912367	1	M+NH4, M+ACN+H	839.7537874	0.93	-	4	Unknown	-	-	Soy drink	2.4
384.0942285	1	-	-	11.11	-	4	Unknown	-	-	Soy drink	2.4
160.1328562	1	-	-	5.14	-	4	Unknown	-	-	Soy drink	2.4
931.7918233	2	-	-	0.90	-	4	Unknown	-	-	Soy drink	2.3
114.1276103	1	-	-	5.14	-	4	Unknown	-	-	Soy drink	2.2
327.2885005	1	-	-	10.94	-	4	Unknown	-	-	Soy drink	2.1

								Postprandial serum		Fasting serum	
**m/z**	**Charge**	**Adducts**	**Neutral mass (Da)**	**Retention time (min)**	**HMDB / NIST ref.**	**Id. level #**	**Name / Class**	**Food source**	**VIP score**	**Food source**	**VIP score**
416.2632955	1	-	-	11.57	-	3	Sub class: Amino acids, peptides, and analogues	-	-	Soy drink	2.0
658.3571811	1	-	-	11.48	-	4	Unknown	-	-	Soy drink	2.0
591.3167511	1	-	-	14.91	-	4	Unknown	-	-	Soy drink	2.0
242.0147031	1	M+H	241.0074272	1.25	HMDB06512	2	3-Mercaptolactate-cysteine disulfide	-	-	Soy drink	2.0
461.904777	1	-	-	1.04	-	4	Unknown	-	-	Soy drink	2.0
591.3162324	1	-	-	7.42	-	4	Unknown	-	-	Soy drink	1.9
639.670691	1	-	-	0.95	-	4	Unknown	-	-	Soy drink	1.9
375.1866099	1	M+2Na-H, 2M+H, 2M+Na	187.0924062	1.30	-	4	Unknown	-	-	Soy drink	1.9
736.9551881	1	-	-	0.99	-	4	Unknown	-	-	Soy drink	1.8
367.2257026	1	-	-	11.45	-	4	Unknown	-	-	Soy drink	1.8
525.2333641	1	-	-	7.33	-	4	Unknown	-	-	Soy drink	1.8
408.0070498	1	M+ACN+Na	343.9912845	0.99	312301	2	Bis(4-fluoro-3-nitrophenyl)sulfone	-	-	Soy drink	1.8
979.168234	1	-	-	1.00	-	4	Unknown	-	-	Soy drink	1.8
135.0552348	1	-	-	4.53	-	4	Unknown	-	-	Soy drink	1.7
535.0339341	1	-	-	8.12	-	4	Unknown	-	-	Soy drink	1.7
180.0652783	1	M+H, M+Na, 2M+H, 2M+Na	179.0580018	5.19	-	4	Unknown	-	-	Soy drink	1.7
105.0334603	1	-	-	5.19	-	4	Unknown	-	-	Soy drink	1.6
878.5851258	1	-	-	8.46	-	4	Unknown	-	-	Soy drink	1.6
458.2012391	1	-	-	6.23	-	4	Unknown	-	-	Soy drink	1.6
466.2568826	2	-	-	6.13	-	4	Unknown	-	-	Soy drink	1.6
220.0733862	1	-	-	1.13	-	4	Unknown	-	-	Soy drink	1.6
147.0440166	1	-	-	4.42	-	4	Unknown	-	-	Soy drink	1.6
944.3619213	1	M+H, M+2H	943.3541334	5.90	-	4	Unknown	-	-	Soy drink	1.6
309.4501147	2	-	-	0.83	-	4	Unknown	-	-	Soy drink	1.6
560.5198846	1	M+H, M+NH4, M+Na	542.486059	5.50	-	3	Direct parent: Carotenes	-	-	Soy drink	1.6
