# Supplementary material for: Identification of Milk and Cheese Intake Biomarkers in Healthy Adults Reveals High Interindividual Variability of Lewis System–Related Oligosaccharides
Source: J Nutr. 2020 Mar 4;150(5):1058–67. doi: 10.1093/jn/nxaa029 (PMC7198293; doi:10.1093/jn/nxaa029)
Supplement: nxaa029_Supplemental_Files [file nxaa029_supplemental_files.zip › G_Pimentel_JNutr_Supplemental_Tables 2_3_4_Revised_21_01_20.pdf]

Identification of milk and cheese intake biomarkers in healthy adults reveals high inter-individual variability of Lewis system related oligosaccharides.
Grégory Pimentel

Online Supplementary Material

SUPPLEMENTAL TABLE 2 Univariate statistical analysis *P* values of serum putative biomarkers of food intake for milk or cheese in healthy adults, with level 1 identification.[1]

A

	1h	2h	4h	6h	6h iAUC	24h
Blood group H disaccharide	0.812	0.879	0.48	0.71	0.633	0.996
Lewis a trisaccharide	0.696	0.101	0.288	0.61	0.549	0.996
Galactonic acid/Gluconic acid	0.696	**0.05**	0.107	0.433	0.064	0.996
Aminoadipic acid	0.915	**0.01**	0.051	0.79	0.064	0.996
Citrulline	0.155	0.099	0.653	0.821	0.246	0.996
Valyl-Threonine	0.377	**0.009**	0.205	0.676	0.068	0.996
Phenylalanyl-Proline	0.599	**0.041**	0.152	0.855	0.068	0.996
Indolelactic acid	0.881	**0.013**	**0.029**	0.388	**0.018**	0.996
Proline	0.406	**0.014**	**0.012**	0.146	**0.008**	0.996

B

	1h			2h			4h			6h			6h iAUC			24h		
	A-B	A-C	B-C	A-B	A-C	B-C	A-B	A-C	B-C	A-B	A-C	B-C	A-B	A-C	B-C	A-B	A-C	B-C
Blood group H disaccharide	-	-	-	-	-	-	-	-	-	-	-	-	-	-	-	-	-	-
Lewis a trisaccharide	-	-	-	-	-	-	-	-	-	-	-	-	-	-	-	-	-	-
Galactonic acid/Gluconic acid	-	-	-	**0.022**	**0.031**	0.920	-	-	-	-	-	-	-	-	-	-	-	-
Aminoadipic acid	-	-	-	**0.000**	**0.004**	0.791	-	-	-	-	-	-	-	-	-	-	-	-
Citrulline	-	-	-	-	-	-	-	-	-	-	-	-	-	-	-	-	-	-
Valyl-Threonine	-	-	-	**0.000**	0.950	**0.000**	-	-	-	-	-	-	-	-	-	-	-	-
Phenylalanyl-Proline	-	-	-	**0.006**	0.719	0.068	0.187	0.708	**0.038**	-	-	-	**0.024**	0.901	**0.038**	-	-	-
Indolelactic acid	-	-	-	**0.002**	0.992	**0.002**	**0.036**	0.867	**0.003**	-	-	-	**0.005**	0.925	**0.002**	-	-	-
Proline	-	-	-	**0.001**	0.717	**0.007**	0.114	0.070	**0.000**	-	-	-	**0.007**	0.499	**0.000**	-	-	-

[1] Kruskal-Wallis sum rank test (*n* = 10 participants) assessing the test foods effect at each time point, based on delta values (subtraction of baseline) and for the 6h-iAUC (A). Post-hoc Conover-Iman pairwise comparison test applied when the Kruskal-Wallis test was significant (B). A, milk intake; B, cheese intake; C, soy drink intake. iAUC, 6 h postprandial incremental area under the curve. Significant differences between foods are in bold ($P < 0.05$ as significant threshold).

SUPPLEMENTAL TABLE 3 Univariate statistical analysis *P* values of urine putative biomarkers of food intake for milk or cheese in healthy adults, with level 1 identification.[1]

A

	0-1h	1-2h	2-4h	4-6h	6h iAUC	6-12h	12-24h
Blood group H disaccharide	0.309	**0.044**	0.051	**0.050**	0.055	0.842	0.807
Galactonic acid/Gluconic acid	0.921	**0.020**	0.065	0.186	0.065	0.790	0.892
Aminoadipic acid	0.921	**0.001**	0.065	0.265	0.065	0.458	0.807
Phenylalanyl-Proline	**0.006**	**0.000**	**0.002**	0.088	**0.002**	0.458	0.922
Indolelactic acid	**0.001**	**0.000**	**0.002**	**0.017**	**0.000**	0.458	0.922

B

	0-1h			1-2h			2-4h			4-6h			6h iAUC			6-12h			12-24h		
	A-B	A-C	B-C	A-B	A-C	B-C	A-B	A-C	B-C	A-B	A-C	B-C	A-B	A-C	B-C	A-B	A-C	B-C	A-B	A-C	B-C
Blood group H disaccharide	-	-	-	0.594	**0.017**	0.076	-	-	-	0.228	**0.017**	0.306	-	-	-	-	-	-	-	-	-
Galactonic acid/Gluconic acid	-	-	-	0.977	**0.014**	**0.013**	-	-	-	-	-	-	-	-	-	-	-	-	-	-	-
Aminoadipic acid	-	-	-	0.000	0.202	**0.001**	-	-	-	-	-	-	-	-	-	-	-	-	-	-	-
Phenylalanyl-Proline	**0.023**	0.281	**0.000**	0.000	**0.009**	**0.000**	**0.039**	**0.020**	**0.000**	-	-	-	**0.016**	**0.022**	**0.000**	-	-	-	-	-	-
Indolelactic acid	**0.003**	0.056	**0.000**	0.000	**0.000**	**0.000**	**0.029**	**0.026**	**0.000**	**0.037**	0.411	**0.004**	**0.001**	**0.000**	**0.000**	-	-	-	-	-	-

[1] Kruskal-Wallis sum rank test (*n* = 10 participants) assessing the test foods effect at each time point, based on delta values (subtraction of baseline) and for the 6h-iAUC (A). Post-hoc Conover-Iman pairwise comparison test applied when the Kruskal-Wallis test was significant (B). A, milk intake; B, cheese intake; C, soy drink intake. iAUC, 6 h postprandial incremental area under the curve. Significant differences between foods are in bold ($P < 0.05$ as significant threshold).

SUPPLEMENTAL TABLE 4 Comparison of three sub-groups of healthy adults showing different Lewis A trisaccharide and blood group H disaccharide levels after milk intake; Kruskal-Wallis and Connover-Imman tests *P* values.[1]

		Serum LeA	Serum BGH	Urine BGH
Krukal-Wallis test (LeA increase vs BGH increase vs No increase)		**0.016**	**0.026**	**0.032**
Conover-Iman pairwise comparison test	LeA increase vs BGH increase	**< 0.001**	**0.001**	**0.006**
	LeA increase vs No increase	**< 0.001**	0.127	0.334
	BGH increase vs No increase	1	**0.031**	**0.004**

[1] Statisticals tests have been conducted using the 6 h postprandial incremental area under the curve from serum or urine samples. LeA was not detected in urine. Three sub-groups of subjects have been identified: subjects presenting an increase in serum Lea (LeA increase, $n = 4$), an increase in serum and urine BGH (BGH increase, $n = 4$), or no increase for either of the two metabolites (No increase, $n = 2$). Significant differences between sub-groups of subjects are in bold ($P < 0.05$ as a significant threshold). LeA, Lewis A trisaccharide; BGH, Blood group H disaccharide.
